# Altered gut microbiome composition by appendectomy contributes to colorectal cancer

**DOI:** 10.1038/s41388-022-02569-3

**Published:** 2022-12-20

**Authors:** Feiyu Shi, Gaixia Liu, Yufeng Lin, Cosmos liutao Guo, Jing Han, Eagle S. H. Chu, Chengxin Shi, Yaguang Li, Haowei Zhang, Chenhao Hu, Ruihan Liu, Shuixiang He, Gang Guo, Yinnan Chen, Xiang Zhang, Olabisi Oluwabukola Coker, Sunny Hei Wong, Jun Yu, Junjun She

**Affiliations:** 1 Department of General Surgery, The First Affiliated Hospital of Xi’an Jiaotong University, Xi’an, Shaanxi China; 2 Center for Gut Microbiome Research, Med-X Institute, The First Affiliated Hospital of Xi’an Jiao tong University, Xi’an, Shaanxi China; 3 Department of High Talent, The First Affiliated Hospital of Xi’an Jiaotong University, Xi’an, Shaanxi China; 4 State Key Laboratory of Digestive Disease, Institute of Digestive Disease and Department of Medicine and Therapeutics, the Chinese University of Hong Kong, Hong Kong SAR, China; 5 Department of Gastroenterology, The First Affiliated Hospital of Xi’an Jiaotong University, Xi’an, Shaanxi China

**Keywords:** Microbiology, Cancer epidemiology

## Abstract

Appendectomy impacts the homeostasis of gut microbiome in patients. We aimed to study the role of appendectomy in colorectal cancer (CRC) risk through causing gut microbial dysbiosis. Population-based longitudinal study (cohort 1, *n* = 129,155) showed a 73.0% increase in CRC risk among appendectomy cases throughout 20 years follow-up (Adjusted sub-distribution hazard ratio (SHR) 1.73, 95% CI 1.49–2.01, *P* < 0.001). Shotgun metagenomic sequencing was performed on fecal samples from cohort 2 (*n* = 314). Gut microbial dysbiosis in appendectomy subjects was observed with significant enrichment of 7 CRC-promoting bacteria (*Bacteroides vulgatus, Bacteroides fragilis, Veillonella dispar, Prevotella ruminicola, Prevotella fucsa, Prevotella dentalis, Prevotella denticola*) and depletion of 5 beneficial commensals (*Blautia sp YL58, Enterococcus hirae, Lachnospiraceae bacterium Choco86, Collinsella aerofaciens, Blautia sp SC05B48*). Microbial network analysis showed increased correlation strengths among enriched bacteria and their enriched oncogenic pathways in appendectomy subjects compared to controls. Of which, *B. fragilis* was the centrality in the network of the enriched bacteria. We further confirmed that appendectomy promoted colorectal tumorigenesis in mice by causing gut microbial dysbiosis and impaired intestinal barrier function. Collectively, this study revealed appendectomy-induced microbial dysbiosis characterized by enriched CRC-promoting bacteria and depleted beneficial commensals, signifying that the gut microbiome may play a crucial role in CRC development induced by appendectomy.

## Introduction

Colorectal cancer (CRC) is one of the most common cancer worldwide [1]. Initiation and progression of CRC involve complex interactions among genetic, epigenetic and environmental factors. Given that hereditary and familial CRC only accounts for 2% to 5% of cases, environmental factors are the key triggers of CRC. Emerging evidence has indicated that gut microbes are an important environmental factor promoting CRC development [2]. Gut dysbiosis has been shown to promote colorectal carcinogenesis in mice [3]. Several individual bacterial species, such as the enterotoxigenic *Bacteroides fragilis* (ETBF), *Fusobacterium nucleatum* and *Peptostreptococcus anaerobius*, could exert carcinogenic effects by inducing direct DNA damage, oxidative damage, and activating oncogenic signaling pathways [3–6].

Recent studies have shown that the appendix plays an important role in maintaining homeostasis and biodiversity of gut microbiome by providing an ideal ecological niche for commensal bacteria and production of immunoglobulin A [7–9]. Considering the key role of microorganisms in gastrointestinal pathophysiology, absence of appendix may result in disruption of microbiome homeostasis, which could potentially influence the risk of developing CRC. In terms of epidemiological evidence, the association of appendectomy with the risk of CRC development has been controversial, and to date no consensus has been attained [10–12]. Although gut microorganisms could be a crucial pivot between appendectomy and risk of subsequent CRC development, the direct contribution of appendectomy and the underlying mechanisms are still largely unexplored.

To uncover the potential association of appendectomy with risk of subsequent CRC incidence, we first performed a population-based epidemiological investigation involving a total of 129,155 subjects to longitudinally assess the clinical connections between appendectomy and the risk of subsequent CRC development. We then conducted a comprehensive fecal shotgun metagenomics sequencing to evaluate taxonomic and functional characteristics of gut microbiome on the cohort with 314 stool samples, sourced from 157 appendectomy cases and 157 matched normal controls without appendectomy. Moreover, we used CRC mouse model with appendectomy to investigate the mechanism of appendectomy-induced colorectal tumorigenesis through causing microbial dysbiosis. The study will enlighten our understanding on the functions of appendix and shed light on the potential role of appendectomy in subsequent CRC development by regulating hemostasis and composition of gut microbiome.

## Results

### Appendectomy increases the cumulative incidence of subsequent CRC development of a population-based longitudinal cohort study

**Fig. 1:**
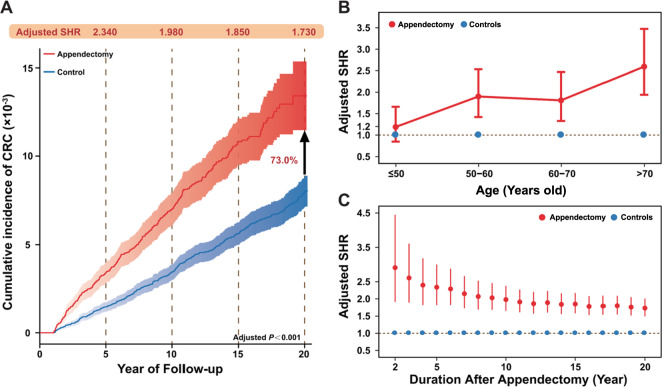
The appendectomy increased the overall cumulative risk of colorectal cancer development based on the longitudinal epidemiological study on Hong Kong Cohort. **A** The cumulative incidence of colorectal cancer (CRC) was increased by 73% in appendectomy cases compared with controls during the 20 years follow-up. **B** The adjusted sub-distribution hazard ratio (SHR) for CRC development stratified by age (≤50 years old, 50–60 years old, 60–70 years old, and >70 years old). **C** The temporal trends of the adjusted SHR for CRC development over the 20 follow-up years in appendectomy cases compared with controls.

We first conducted a large epidemiological study to explore the association between appendectomy and subsequent CRC risk. A total of 43,976 appendectomy cases and 85,179 age- and gender- non-appendectomy controls were included in further analysis (Fig. S1A). Basic characteristics of these participates were shown in Table S1. During a total of 1,401,020 person-years follow-up time, the CRC incidence was 73.1 (95% CI: 65.0–81.2) per 100,000 person-years in appendectomy group, whereas CRC incidence in controls was 39.7 (95% CI: 35.8–43.7) per 100,000 person-years (Table S2). The overall risk of subsequent CRC development increased by 73.0% in appendectomy cases with sub-distribution hazard ratio (SHR) 1.730 (95% confidence interval (CI) 1.490–2.010) compared with controls (Fig. 1A and Table S2). Subgroup analysis further indicated that appendectomy-treated subjects with aged > 50 years had significantly higher risk for CRC development (SHR 2.020 and 95% CI 1.710–2.396), as compared to appendectomy cases with aged ≤50 years (SHR 1.190 and 95% CI: 0.848–1.660) (Fig. 1B, Table S2 and Fig. S2). In addition, appendectomy cases had significantly higher risk for CRC development in proximal colon (SHR 2.210 and 95% CI: 1.640–2.990) compared with distal colon (SHR 1.670 and 95% CI 1.290–2.150) and rectum (SHR 1.570 and 95% CI 1.210–2.102), respectively (Table S2 and Fig. S2). During the entire follow-up period, the adjusted SHR for CRC development decreased over time, while the risk of CRC in appendectomy cases remained significantly higher than control individuals (Fig. 1C). Subgroup analyses showed the consistent results (Fig. S3).

### Compositional shift of gut microbiome in appendectomy cases

**Fig. 2:**
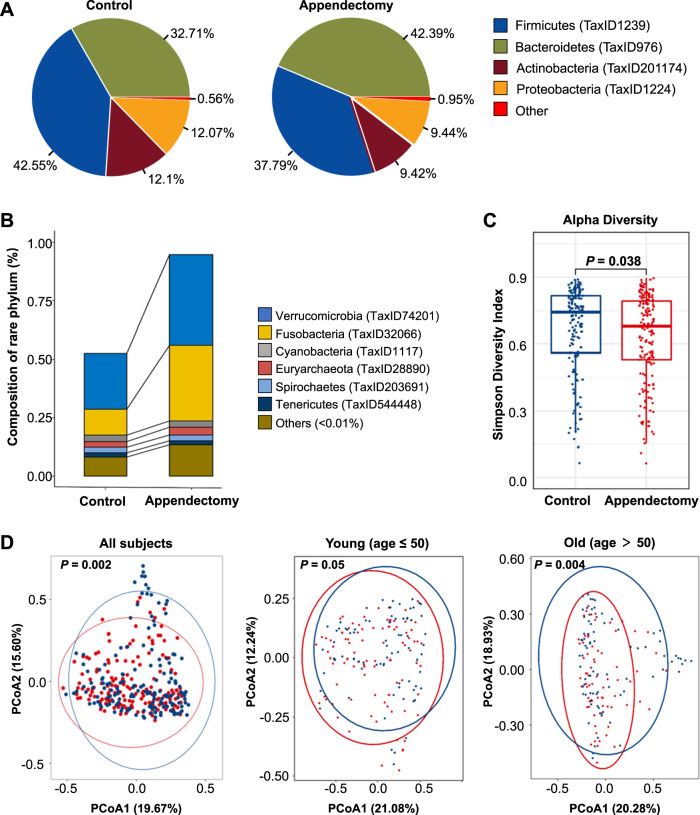
The appendectomy altered the microbial community profile. **A** The microbial composition at phylum level in appendectomy and control groups. Phylum with relative population above 1% was denoted as abundant, while “Other” was denoted as the sum of rare phyla with relative population less than 1%. **B** Rare phylum composition in appendectomy and control subjects. “Other” represented the sum of phyla with relative population less than 0.01%. **C** Alpha diversity for Simpson index at genus level. **D** Principal coordinates analysis for all subjects, young subjects (age ≤ 50), and old subjects (age > 50). Red stands for the appendectomy group, while blue represents the control group.

Given the positive clinical association between appendectomy and CRC development, we next investigated the potential contribution of gut dysbiosis induced by appendectomy to the risk of CRC. Gut microbiome profiling was performed in 314 fecal samples from 157 appendectomy cases and 157 normal controls by shotgun metagenomic sequencing (Fig. S1B). Demographic and clinical characteristics of these participants were shown in Table S3. High-quality sequencing reads (mean 15 GB per sample) with an average of 34,168,657 paired reads per sample were obtained after filtering for microbial taxonomic classification (Table S4). Significantly shift in microbial composition was observed at phylum level with relative abundance ≥ 1% (Fig. 2A). In particular, *Fusobacteria* phylum was markedly enriched in the appendectomy group (Fig. 2B). Subjects with appendectomy had a lower microbial alpha diversity (Simpson index of genera with relative abundance >1%) in their gut microbiome compared to normal controls (*P* < 0.05) (Fig. 2C). Beta diversity analysis showed separated clusters between appendectomy cases and normal controls (PERMANOVA, *P* < 0.05; Fig. 2D). To assess whether the microbial signature is influenced by the sampling time, we examined the microbiome in specimens collected 6 to 24 months after appendectomy. The alpha and beta diversities were similar among specimens collected at different timepoints after appendectomy (6 months, 6–12 months, 12–18 months, 18–24 months, and 24 months after appendectomy) (Fig. S4A, B), suggesting that the microbial community changes caused by appendectomy could be persistent for over 2 years. Considering the impact of age on gut microbiome, β-diversity analysis on older (> 50 years) and younger subgroups (≤50 years) was conducted, respectively. Older subjects showed significant difference in microbiome composition between appendectomy cases and controls (PERMANOVA, *P* = 0.004), while no significant difference was observed among younger subjects (Fig. 2D). These observations suggested that appendectomy leads to alteration in gut microbiome composition, especially for elder individuals.

### Appendectomy induces enrichments of CRC-associated species in gut

**Fig. 3:**
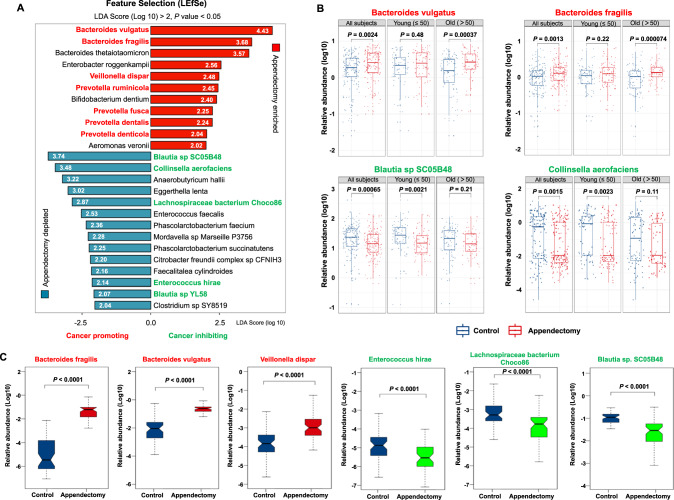
Differential abundance of bacterium species in appendectomy patients compared to control subjects. **A** Bar plot for the differential abundant species identified by LEfSe. Red bars stand for the enriched species in appendectomy patients, while blue bars represent the depleted species in appendectomy subjects. Species that were reported to promote cancer were marked in bold red fonts, while the cancer-inhibiting species were marked in bold green fonts. **B** The relative abundance for representative appendectomy enriched species, *Bacteroides vulgatus* and *Bacteroides fragilis*; and appendectomy-depleted species, *Blautia sp SC05B48* and *Collinsella aerofaciens* in all subjects, young and old subgroups are compared and shown as box plot. **C** The validation of identified appendectomy-enriched and depleted bacteria species including *Bacteroides fragilis, Bacteroides vulgatus, Veillonella dispar, Enterococcus hirae, Lachnospiraceae bacterium Choco86, Blautia sp. SC05B48*) was shown by using targeted qPCR in appendectomy cases and control subjects.

We then extended our microbial analysis by evaluating the difference in gut microbiome at species level. 25 bacterial species were identified with significant difference in abundances (11 enriched and 14 depleted) in appendectomy cases compared with control subjects (Fig. 3A). Among the 11 enriched species, 7 were reported as CRC- or cancers-associated bacteria (*Bacteroides vulgatus*, *Bacteroides fragilis*, *Villanelle dispar*, *Prevotella ruminicola*, *Prevotella fusca*, *Prevotella dentalis*, and *Prevotella denticola*) [6, 13–18]. While 5 were reported as protective bacteria (*Blautia sp SC05B48*, *Collinsella aerofaciens*, *Lachnospiraceae bacterium Choco86*, *Enterococcus hirae*, *Blautia sp YL58*) [15, 19–22] among the 14 depleted species (Fig. 3A and Figs. S5, S6). In particular, the top two enriched bacteria in appendectomy subjects, *B. vulgatus and B. fragilis*, were found to have age-specific correlation, in which they were significantly enriched only in the older subgroup (both *P* < 0.001) (Fig. 3B). The top two depleted bacteria in appendectomy, *B. sp SC05B48* and *C. aerofaciens*, also showed age-specific correlation with significant depletion in the younger subgroup (both *P* < 0.01) (Fig. 3B). Such changes in bacterial abundances (*B. vulgatus, B. fragilis, B. sp SC05B48* and *C. aerofaciens*) were sustained for over 2 years after appendectomy (Fig. S7A). Moreover, the virulence factor gene *pks* was significantly increased (*P* < 0.05) in appendectomy cohort compared to normal controls (Fig. S7B). To further validate the abundance of differentially enriched bacteria identified by our metagenomic analyses, we performed qPCR of 3 representative enriched (*B.fragilis, B. vulgatus, V. dispar*) and depleted bacteria (*E. hirae, Lachnospiraceae bacterium Choco86, Blautia sp. SC05B48*) in appendectomy cases. The results showed a consistent distribution of differential enriched bacterial species as metagenomic analyses (Fig. 3C and Fig. S8). Collectively, these observations consistently indicated that alterations in gut microbiome after appendectomy could be pathogenic with enrichments of several CRC-associated bacteria and depletion of beneficial commensals.

### Microbiome ecological network is altered after appendectomy

**Fig. 4:**
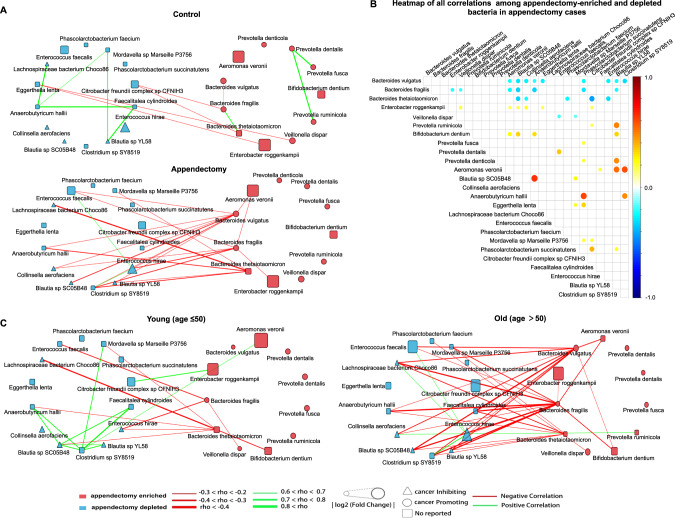
Correlation analysis of differential abundant bacteria species. **A** Correlation network among differential abundant species (enriched/depleted) selected by LEfSe in appendectomy and control groups. **B** Heatmap for all stronger relationships in appendectomy cases among appendectomy-enriched/depleted bacteria. The node size in the heatmap represented Spearman’s rank correlation coefficient. **C** Subgroup analysis (age ≤50/ age > 50 years) of associations in appendectomy cases among selected bacteria with correlation coefficient *rho* below −0.2 or above 0.6 are shown. Correlations were calculated by Spearman’s rank correlation. The node size in the networks represents log2 (Fold Change). The red lines represent co-exclusion correlation, and green lines represent co-occurrence association. Species that were reported to promote cancer were marked in red fonts, while the cancer-inhibiting species were marked in blue fonts.

To gain insights into ecological networks among bacteria, we investigated correlations between enriched and depleted species in appendectomy subjects by Spearman’s Rank correlation analyses. We found that enriched and depleted bacteria separately formed their own networks with negative correlation with each other (Fig. 4A, B). While the number of negative correlations was significantly increased in the appendectomy group at the ratio of 20:4 ratio, contrasting to the ratio of 2:9 for positive correlations (Fig. 4A, B, Tables S5,S6). In particular, we identified that *B. fragilis and B. vulgatus*, two of appendectomy-enriched species and the CRC-associated pathogen [14, 16], was the centrality of the network of enriched bacteria and had the strongest negative correlation with the network of depleted bacteria (Fig. 4A, C, Table S7). Whereas appendectomy-depleted bacteria showed significant synergistic relationships (Fig. 4A, C), possibly due to the protective effects of beneficial bacteria in the gut microenvironment. Meanwhile, in consistent with the epidemiological results, we observed more negative correlations among appendectomy-enriched and -depleted species in older subjects than younger subjects (Fig. 4C). The appendectomy-enriched bacteria, including *B. fragilis* and *B. vulgatus*, showed increased negative correlations with commensal bacteria in the older subgroup, while protective bacteria such as *Blautia sp. SC05B45* and *Blautia sp. YL58* had negligible influence. Moreover, correlations among appendectomy-depleted bacteria were more positive in the younger subgroup than in the older subgroup. Taken together, the change in gut microbial ecology after appendectomy indicated that appendectomy could impact interactions among gut microbes which may contribute to CRC development in appendectomy cases, especially in aged subjects.

### Functional features of gut microbiome are altered after appendectomy

**Fig. 5:**
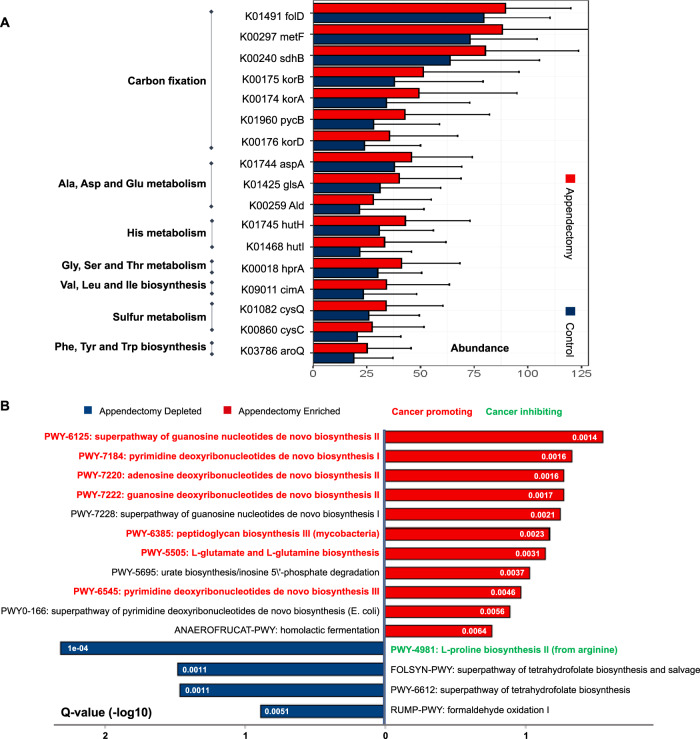
Microbiome functional capacity changes in appendectomy patients. **A** Appendectomy-induced alteration of abundance in microbial genes was summarized by KEGG pathway modules. The genes enriched in appendectomy compared to normal control were evaluated for gene relative abundance profiles (Wilcoxon rank-sum test followed by FDR correction using “Benjamini–Hochberg” methods, *q* < 0.1), and those with mean relative abundance >1% were shown. The relative gene abundance was shown as bar plots by averaging all appendectomy samples (*n* = 157). **B** Bar plot for Pathway Enrichment Analysis by Humann2 with Meta-Cyc database. The red bar standard for appendectomy-enriched pathways compared with control group, whist the blue bar represented the appendectomy-depleted pathways. Pathways reported to promote cancer were marked in red fonts, and the cancer-inhibiting ones were marked in green fonts.

The contribution of gut microbial dysbiosis to pathological conditions could also be mediated by their functional capabilities. Functional changes at species-level were characterized by Kyoto Encyclopedia of Genes and Genomes (KEGG) database, and a total of 227 KEGG orthology genes (KO genes) showed significant enrichments in appendectomy group, compared to healthy controls (adjusted *P* < 0.1). We then examined abundances of microbial genes to assess the functional roles of gut microbes in appendectomy-associated CRC risk. Functional pathways including amine acid metabolism, carbon fixation, sulfur metabolism, and aromatic amino acid metabolism were significantly different in appendectomy cases (Fig. 5A), of which all of them were reported to be associated with CRC development [23–26]. Changes in the functional microbial pathway were examined using HUMAnN2, which enables robust profiling on microbial pathway abundance in distinct metagenome functional contents. We identified 15 metabolic pathways with differential abundances between appendectomy cases and controls (*q* < 0.2). Microbiome in appendectomy cases was dominated by biosynthesis pathways of deoxyribonucleotides (pyrimidine, adenosine, guanosine), peptidoglycan, L-glutamate L-glutamine, and pyrimidine deoxyribonucleotides (*P* < 0.01, for all), which were all reported as cancer-promoting metabolic pathways [27–29]. Moreover, the biosynthesis of L-proline from arginine was known to be cancer-inhibitory and was found to be depleted in appendectomy cases [26] (*P* < 0.0001) (Fig. 5B).

### Appendectomy promotes colorectal tumorigenesis through altering microbial composition and inducing intestinal barrier dysfunction in mice

**Fig. 6:**
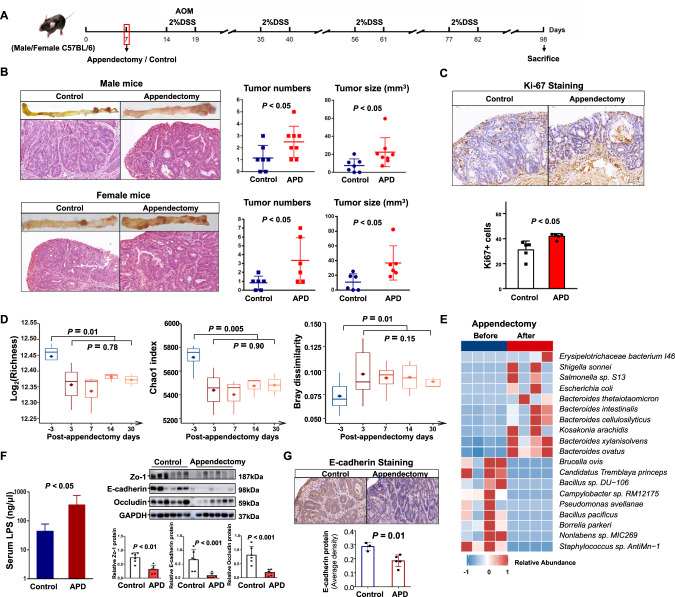
Appendectomy promotes colorectal tumorigenesis through altering microbial composition and inducing intestinal barrier dysfunction in mice. **A** Schematic diagram of mice experiment. Appendectomy and control were performed in a AOM/DSS-induced CRC mouse model. **B** Representative images of colorectal tumors from mice with control or appendectomy (top) and H&E staining of tumors (bottom). The tumor load was evaluated by tumor number and tumor size (mm^3^). **C** Immunohistochemistry staining of Ki-67^+^ cells in colon tumors of appendectomy or control mice. **D** Gut microbial alpha diversity by richness and Chao1 and the beta-diversity measured by dissimilarity using bray-cutis distance in stool samples collected at baseline (3 days before appendectomy), and 3, 7, 14, 30 days after appendectomy. **E** Differentially enriched gut bacteria before and after appendectomy in mice. **F** Serum lipopolysaccharides concentration and Western blot analysis of Zo-1, Occludin, and E-cadherin in colon of Appendectomy and control mice. Band intensity was measured by Image J and the ratio of each band was normalized to the corresponding GAPDH. **G** The E-cadherin protein expression in colon tumor from appendectomy and control mice.

To confirm the influence of appendectomy on microbial dysbiosis and colorectal tumorigenesis, we performed appendectomy or sham in a carcinogen-induced CRC mouse model (Fig. 6A). The results showed that tumor number (*P* < 0.05) and tumor size (*P* < 0.05) were significantly higher in both male and female mice with appendectomy as compared to control mice (Fig. 6B), while no significant changes in body weight was observed (Fig. S9A). Histological examination of colon tumor sections confirmed that appendectomy induced cell proliferation as evidenced by significantly increased Ki-67 positive cells (Fig. 6C).

Fecal samples of mice at different time points including baseline (3 days before appendectomy), and 3, 7, 14, 30 days after appendectomy, were subjected to metagenome sequencing for investigating the effects of appendectomy on gut microbiome. Compared to baseline, the microbial alpha diversity (Richness and Chao1) was significantly decreased 3 days after appendectomy (Fig. 6D). Significant difference in beta-diversity as measured by dissimilarity using bray-cutis distance was also observed between baseline and 3 days after appendectomy (Fig. 6D). In contrast, the microbial diversity (alpha and beta-diversity) was similar among fecal samples collected 3, 7, 14 and 30 days after appendectomy (Fig. 6D), suggesting the microbiome changes could persist after appendectomy. Moreover, some pathogenic bacteria, including *Bacteroides intestinalis* and *Shigella sonnei*, were enriched after appendectomy (Fig. 6E and Table S8).

We further examined the intestinal barrier function, which is known to be greatly impaired in colorectal tumorigenesis, in mice receiving appendectomy. Serum lipopolysaccharides concentration was significantly elevated in mice with appendectomy compared to control mice (Fig. 6F). In keeping with this, the expressions of tight junction proteins Zo-1 (*P* < 0.01), Occludin (*P* < 0.001), and adherent junction protein E-cadherin (*P* < 0.001) were all significantly reduced in the colon of appendectomy mice (Fig. 6F), implying a remarkable impairment of intestinal barrier function in appendectomy mice. Moreover, the protein expression of E-cadherin was decreased in tumor tissues of post-appendectomy mice compared to control (*P* = 0.01, Fig. 6G). Accordingly, the E-cadherin inhibited oncogenic Hippo signaling [30], was activated in tumor tissue of post-appendectomy mice compared to the control mice as identified by RNA sequencing (Fig. S9B). Collectively, these results indicated the CRC-promoting role of appendectomy was associated with gut dysbiosis through impairing intestinal barrier function and downregulated E-cadherin signaling.

To verify the roles of gut bacteria in promoting CRC after appendectomy, we depleted the gut microbiome by antibiotic cocktail treatment in both male and female mice (Fig. S10A). The depletion of gut microbiome by antibiotics was confirmed by qPCR on mouse fecal samples (Fig. S10B). No difference in body weight was found between control and appendectomy groups with antibiotic treatment (Fig. S10C). Appendectomy could not affect CRC development in microbiome-depleted mice, which had similar levels of tumor number and tumor size as in antibiotic-treated mice with sham, regardless of their gender (Fig. S10D). These findings corroborated that intestinal bacteria dysbiosis caused by appendectomy play a key role in CRC tumorigenesis after appendectomy.

## Discussion

In this study, we observed a significantly increased risk of CRC development following appendectomy based on the population-based cohort study involving 129,155 subjects (HR 1.730 and 95% CI: 1.490–2.010) with 20-year follow-up. We also demonstrated that appendectomy cases with age > 50 years old had higher risk of CRC development over the whole 20 years of follow-up, compared to younger subjects (age ≤ 50 years). Our findings suggested that appendectomy contributes to the increased risk of CRC development, and aged subjects with history of appendectomy are associated with higher risk of subsequent CRC.

Despite the strong association between appendectomy and the risk of subsequent CRC development as demonstrated by our epidemiological study, the effect varies remarkably with exposure time of appendectomy. In particular, the risk of CRC development among appendectomy cases is increased within the first two years after operation but gradually decreased afterwards. Given the role of appendix as a lymphoid organ to maintain dynamic equilibrium of the gut microenvironment, the findings in this study proposed that appendectomy could possibly induce durative compositional change in the gut microbiome at least within two years, which may be gradually compensated by time. Notably, some studies reported the correlation between appendicitis and increased risk of CRC. Whereas in individuals with appendectomy treatment, the possibility of appendicitis-associated CRC is eliminated following appendix removal, and thus in long-term follow up, appendectomy plays a critical role in contributing to CRC.

To investigate how appendectomy impacts the gut microbiome to contribute to CRC development, shotgun metagenomic sequencing was performed on 314 fecal samples from 157 appendectomy cases and 157 non-appendectomy controls. Of note, to avoid the potential influence of appendicitis itself on the gut microbiome, fecal samples were collected from 6 months up to 2 years after appendectomy. Our metagenomic analysis revealed that appendectomy significantly induced gut dysbiosis with greater predominance in older subjects. This finding was consistent with the increased risk of CRC development in appendectomy cases especially in aged patients. Decreased alpha-diversity and altered beta-diversity were also demonstrated in appendectomy cases, therefore supporting the concept that the appendix serves as a microbial reservoir for commensal bacteria or “safe house role” [7, 8]. Microbial diversity represents the community complexity of gut microbiome, and a higher diversity is known to be associated with healthier situation [31]. Whereas low bacterial diversity has been linked to various intestinal diseases including CRC [13, 32]. Our results reported a gradual compositional shift of the gut microbiome with decreased microbial diversity, enrichment of pathogenic microbes and depletion of protective microbes after appendectomy, thus confirming that appendectomy could induce gut dysbiosis.

At species level, we identified 7 bacteria that were enriched in appendectomy cases including *B. vulgatus, B. fragilis*, *V. dispar, P. ruminicola, P. fusca, P. dentalis*, and *P. denticola*. Among these enriched bacteria, *B. vulgatus* is the most significantly enriched species, which was reported to be involved in colorectal tumorigenesis [16] and inflammatory bowel disease [33]. *B. fragilis*, especially ETBF, was widely reported to promote CRC tumorigenesis by directly inducing DNA damage and Th17 cell immune response of epithelial cells [6, 18]. ETBF could also promote biofilms formation in colorectal mucosa by recruiting other bacteria to cause leucocyte chemotaxis, inflammation, and colon tumorigenesis [14]. We found that both *B. vulgatus* and *B. fragilis* were significantly enriched in older subjects with history of appendectomy, which partially explained our epidemiological observations that appendectomy increased CRC risk after appendectomy was more frequently occurred in older patients. *Veillonella* genus was reported to be associated with Crohn’s disease [13] of which *Veillonella spp*. could stimulate cytokine induction and oncogenic p38 MAPK activation in TLR4-dependent features to promote inflammation [34]. Collectively, these findings suggested that enrichments of potential pathogenic bacteria after appendectomy could be associated with colorectal tumorigenesis. On the other hand, 5 protective bacteria (*Blautia sp SC05B48, Lachnospiraceae bacterium Choco86, Blautia sp YL58, Collinsella aerofaciens*, and *Enterococcus hirae*) were depleted in appendectomy cases. Members of *Lachnospiraceae* family including *Blautia, Lachnospiraceae Bacterium Choco86* were reported to protect against CRC development by conferring colonization resistance to CRC-associated oral taxa [15]. The mucosal abundance of *Lachnospiraceae* genus was significantly lower in CRC patients and inversely associated with abundances of CRC-associated bacterial operation taxonomic units [15]. Moreover, *Blautia spp*. and *Collinsella aerofaciens* could produce short chain fatty acids, which play an important role in protecting against inflammation and CRC [19–21]. While *E. hirae* was also shown to possess anti-inflammatory properties [22]. Taken together, depletions of these protective bacteria in appendectomy patients further indicated the contribution of gut dysbiosis following appendectomy to colorectal tumorigenesis. Further investigations on the implications of microbiome functional dysbiosis in appendectomy patients are needed for deeper understanding of appendectomy-induced CRC development.

A microbial community consisting of biofilm-forming bacteria is known to have virulence properties and capacities to promote cancer development by metabolism modulation. Thus, a microbial network could reflect disease-specific microenvironment. We observed that interactions among gut microbes were significantly affected by appendectomy (Fig. 4A, B). Co-exclusive interactions among appendectomy-enriched and -depleted bacteria were enhanced as compared to control subjects. Meanwhile, the synergistic effect among commensal bacteria was weakened in appendectomy patients, which could be caused by the antagonistic effects from pro-carcinogenic taxa, in particular *B. fragilis and B. vulgatus* (Fig. 4A, B). Depletion of protective taxa may also reduce their suppressive effects on appendectomy-enriched potential pathogenic bacteria. In turn, enrichments of pro-inflammation and pro-carcinogenic taxa could further restrain abundances of commensal bacteria. Such changes in the microbial compositional networks have been previously reported in CRC patients [35]. Moreover, the co-exclusive correlations among appendectomy-enriched (e.g., *B. Fragilis, B. vulgatus*) and appendectomy-depleted bacteria (e.g., *Blautia* and *Lachnospiraceae bacterium Choco86*) were significantly stronger in aged patients compared to younger patients (Fig. 4C). Thus, the changes of microbial networks following appendectomy could partially explain how gut dysbiosis caused by appendectomy contributes to the increased risk of subsequent CRC development, particularly in aged patients.

Metabolites produced by gut microbes are essential to health and disease of host. Here, we identified functional shifts in the gut microbiome that may reflect compositional differences between appendectomy patients and normal controls. Microbial genes enriched in appendectomy were involved in carbon fixation, sulfur metabolism, and aromatic amino acid metabolism (Fig. 5A). These functional metabolism pathways were reported to be associated with CRC formation [23–26]. Our analysis also revealed that meta-cyc pathway shifted in appendectomy cases relevant to normal controls (Fig. 5B). Pro-inflammatory pathways were also enriched in appendectomy cases as well as the biosynthesis of peptidoglycan (a bacterial cell wall polymer), which was known to induce intestinal inflammation [27]. The super pathway of adenosine nucleotides de novo biosynthesis was also enriched in appendectomy cases. Chronic accumulation of adenosine, an ancient extracellular signaling molecule, is associated with increased onset of neoplasia via triggering immune suppression [28] and has a crucial role in cancer development [29]. Collectively, enrichments of these metabolism pathways are closely associated with carcinogenic microenvironment in appendectomy patients, thus highlighting their potential contribution to colorectal tumorigenesis. Nevertheless, further investigations on the implications of microbiome functional dysbiosis after appendectomy are needed for deeper understanding of appendectomy-induced CRC development.

We further confirm the role of appendectomy in promoting colorectal tumorigenesis in CRC mouse model. Our results showed that appendectomy promoted CRC development in mice is consistently associated with gut dysbiosis. The difference in diversity and abundance of mice intestinal microflora persist during tumor induction period (ranging from 3 to 30 days) after appendectomy. Certain pro-inflammation bacteria such as *Bacteroides intestinalis*, *Shigella sonnei* were found to increase after appendectomy, similar to what we have detected in human samples. It is noteworthy that depletion of intestinal microbiota resulted in similar tumor size and number in mice independent of appendectomy, indicating the gut microbiota playing the crucial role in subsequent CRC development induced by appendectomy. Furthermore, gut dysbiosis could induce intestinal barrier dysfunction, which may contribute to translocation of pathogenic microbes and their derivatives into the circulation for inducing CRC development. In keeping with this, we identified the decrease in expressions of tight junction proteins (Zo-1 and Occludin) and adherent junction protein (E-cadherin) in colon tissue of appendectomy-treated mice, thus implicating the occurrence of disrupted intestinal barrier function. In addition, we found decreased E-cadherin expression in tumor tissues of post-appendectomy mice. The downregulation of E-cadherin has been observed in various epithelial cancers such as colorectal, breast, cervical, and ovarian cancer [36]. Loss of E-cadherin in epithelial cells due to gut microbial dysbiosis may also activate multiple oncogenic signaling pathways [37–39]. Consistently, we found the increased oncogenic Hippo signaling pathway in tumors of mice with appendectomy. Therefore, the loss of E-cadherin in post-appendectomy mice not only contributes to impaired gut barrier but also colorectal tumorigenesis.

In conclusion, our population-based longitudinal study identified a 73.0% increase in CRC risk among appendectomy cases compared to controls throughout 20 years of follow-up. Mechanistically, appendectomy caused gut microbial dysbiosis with significant enrichment of cancer-promoting bacteria and depletion of beneficial commensals, and altered the correlations among bacteria and their functional pathways, which contribute as least in part to the appendectomy-associated increased CRC development. This study provides insights on the function of appendix by regulating hemostasis and composition of gut microbiome, and our findings thus suggest surgeons to more cautiously consider the necessity of appendectomy to reduce subsequent CRC development.

## Materials and methods

### Study design and participants

A large retrospective cohort epidemiological study was established to explore the association between appendectomy and subsequent CRC risk. The population-based longitudinal cohort was recruited from a large territory-wide health care database (Clinical Data Analysis and Reporting System, CDARS) in Hong Kong. The CDARS [40, 41] is operated by the Hong Kong Hospital Authority, and contains medical records of all public hospitals, which serve more than 90% of medical services of Hong Kong with 7.3 million populations. We identified 61,396 individuals underwent appendectomy from January 2000 to December 2018 (Fig. S1A). The exclusion criteria included: (1) patients with the age ≤18 years or >95 at enrollment (*n* = 8260); (2) Patients with incidental appendectomy or with any malignant diseases or inflammatory bowel disease history at enrollment (*n* = 3663); (3) Patients with appendiceal neoplasms (*n* = 143); (4) Patients with the diagnosis of CRC within 1 year after appendectomy (*n* = 1470). For the control group, we randomly identified a pool of individuals (*n* = 354,982) without appendectomy between January 2000 and April 2020 from the total population register of CDARS. After exclusion based on the same criteria, a total of 288,646 individuals were included in further analysis. For each case of appendectomy, we selected two matched referential individuals from the pool based on the year of birth, gender and comorbidities (Table S1). All study subjects were followed up from recruitment to the date of CRC diagnosis, death, or until April 1st, 2020. Totally, 43,976 appendectomy cases and 85,179 non-appendectomy controls from the CDARS were included in the analysis (Fig. S1A).

We recruited another cohort (*n* = 513) of 253 appendectomy individuals who underwent laparoscopic appendectomy for histologically-confirmed diagnosis of appendicitis, and 260 healthy individuals without appendectomy at the first affiliated hospital of Xi’an Jiaotong University (Fig. S1B). Fecal samples were collected between August 2019 and April 2020 from these subjects by individuals at home or hospital followed by immediate freezing in dry ice, and deep frozen in −80 °C within 2 h of stool collection for long term storage. Fecal samples from appendectomy cases were collected at postoperative period ranging from 6 months to 2 years. The exclusion criteria for these subjects include: (1) Subjects using hormones, antibiotics, probiotics, or traditional Chinese medicine within three months before fecal samples collection; (2) Subjects with inflammatory bowel disease, pre-cancerous or malignant lesions confirmed in a screening colonoscopy examination; (3) Subjects with history of malignant diseases or autoimmune disease. Based on the above exclusion criteria, a total of 314 individuals (157 appendectomy cases and 157 age- and gender- matched normal controls) were finally included in this cohort and their fecal samples were subjected to shotgun metagenomic sequencing (Fig. S1B).

All subjects signed the informed consents prior to the sample collection. This study was conducted in accordance with the Declaration of Helsinki, and approved by the ethics committee of the First Affiliated Hospital of Xi’an Jiaotong University and the Joint Clinical Research Ethics Committee of the Chinese University of Hong Kong and the Hospital Authority New Territory East Cluster. All clinical data obtained from the CDARS databases were de-identified before analysis.

### Metagenomics sequencing analysis

The fecal DNA of each subject extracted was subjected to library construction following the manufacture’s instruction. Shotgun metagenomics sequencing was conducted on Hiseq 2500 platform (Illumina) as 2 × 150 bp paired-end reads with an average data size of 15 Gb per sample. Quality control of the raw reads was performed using Trimmomatic V0.39 [42] for low quality base trimming (quality score < 30) and short-length reads (<100 bp) removal using the following parameters: SLIDINGWINDOW:4:30 and MINLEN:100. Contaminated reads from host were discarded by mapping against human genome using Bowtie2 (version 2.3.5) with default ‘-very-sensitive -dovetail’ parameters. An average of 0.1% of the reads from the 314 samples were classified as human reads and removed. Finally, the high-quality reads with average size of 50 million were used for further analyses. While fecal samples from mice were processed following the similar procedure and parameters as above, with the exception that contaminated reads in mice metagenomic sequencing data were filtered by mapping against mouse genome.

### Appendectomy in CRC mouse model

To investigate the roles of appendectomy in colorectal tumorgenesis, male and female C57BL/6 mice (5 weeks old) were pretreated individually with ordinary drinking water or a cocktail of broad-spectrum antibiotics (ampicillin (0.2 g/L), vancomycin (0.1 g/L), neomycin (0.2 g/L), and metronidazole (0.2 g/L)) in drinking water for 14 days to deplete most intestinal bacteria. These normal and antibiotic-treated mice were randomly subjected to either surgical removal of appendix lymphoid (appendectomy group) or abdominal incision (control group). All post-surgery mice in both control and appendectomy groups were blindly intraperitoneally injected with a single dose of 10 mg/kg AOM (azoxymethane, Aladdin, Shanghai, China), followed by 4 cycles of 2% DSS (dextran sulfate sodium, MP Biomedicals, Solon, Ohio, USA) administration to induce colorectal tumors. Additional once cocktail antibiotics were administrated for antibiotic-treated mice to minimize the recovery of intestinal bacteria during tumor induction.

To evaluate the effect of appendectomy on intestinal microbial community and its dependence on post-appendectomy time, fecal samples of CRC model mice without antibiotic treatment were collected before appendectomy and at 3, 7, 14, 30 days after appendectomy for metagenomic sequencing. At the end of the experiment, all mice were anaesthetized and sacrificed. Colons of mice were longitudinally opened and rinsed with PBS. Total number of tumors in colon were recorded. Size of each tumor was measured using previous published formula [43]. The total protein was further extracted from the entire colon tissue. All animal experiments were approved by the Institutional Animal Care and Use Committee of Xi’an Jiao tong University.

### Statistical analysis

Propensity score matching was used to analyze and adjust the distribution balance of covariates between the two groups. For each individual in longitudinal cohort study, we calculated person-years of follow-up until the date of CRC diagnosis, death, or April 1st, 2020. We used competing risk model to estimate and calculate SHR and 95% CI for CRC development after adjusting for confounding variables. The subgroup analyses were categorized based on age, gender and tumor location. Furthermore, we established curve of SHRs-time to assess the time trend of CRC risk by calculating SHRs for CRC incidence at different follow-up years after appendectomy. Sensitivity analyses were conducted by (1) calculating SHRs for CRC development using the dataset without trimming of extreme data (Table S9); (2) excluding individuals who were diagnosed with CRC within 3 or 5 years after appendectomy (Table S10). *P* < 0.05 (two-sided) was considered to be statistically significant. All epidemiological statistical analyses were performed using R Project for Statistical Computing software (version 4.0.2).

In metagenomics sequencing analysis, the difference in microbial community composition was evaluated by pairwise PERMANOVA tests with false discovery rate (FDR) adjustments based on bray distance. LEfSe (Linear discriminant analysis Effect Size) algorithm was used to determine the differentially abundant species and significantly enriched/depleted pathways in the appendectomy group by pairwise comparisons with the control. A corrected *P* < 0.05, by FDR, was considered to be significantly different. The KO genes significantly enriched in appendectomy was identified by comparing to control using Mann–Whitely *U* tests with FDR corrected *P* < 0.1.

## Supplementary information

Supplementary methods

Supplementary Figure 1

Supplementary Figure 2

Supplementary Figure 3

Supplementary Figure 4

Supplementary Figure 5

Supplementary Figure 6

Supplementary Figure 7

Supplementary Figure 8

Supplementary Figure 9

Supplementary Figure 10

Supplementary Table 1-11

## Data Availability

Most data supporting the findings of this study are available from the main text of the article and its supplementary materials. RNA sequencing data have been deposited in the Sequence Read Archive (SRA) under the accession code PRJNA906334.
