# Supplementary material for: Altered gut microbiome composition by appendectomy contributes to colorectal cancer
Source: Oncogene. 2022 Dec 20;42(7):530–40. doi: 10.1038/s41388-022-02569-3 (PMC9918431; doi:10.1038/s41388-022-02569-3)
Supplement: Supplementary file 1 — Supplementary methods [file 41388_2022_2569_MOESM1_ESM.docx]

**Supplementary Methods**

**Details of propensity score matching**

Propensity score matching was performed using R V.4.0.2 (R Foundation for Statistical Computing) statistical software and MatchIt package V.4.1.0. We established a logistic regression model to calculate the propensity score conditional on the 17 aforementioned covariates to represent the probability of appendectomy. Before PS matching, we trimmed the data to exclude the bias from extreme ends of the PS distribution. Based on the work of Sturmer T et al, there is a reduction of bias with restriction up to the 2.5-97.5 level, but further restriction cannot further decrease bias. We created 40 categories of 2.5% each for the PS, and trimmed the first and 40th categories. Appendectomy patients were matched to non-appendectomy individuals in a 1:2 ratio without replacement using a greedy distance-based matching algorithm within a calliper width of 0.02. We assessed the balance of the covariates between the two groups by using absolute standardized difference (ASD). The ASD was calculated by the formula in the previous literature.

For continuous variables, the ASD is defined as

$$ASD=\frac{\overline{x}_{\text{treatment}}-\overline{x}_{\text{control}}}{\sqrt{\frac{s_{\text{treatment}}^{2}+s_{\text{control}}^{2}}{2}}}$$

where $\overline{x}_{\text{treatment}}$ and $\overline{x}_{\text{control}}$ denote the sample mean of the covariate in treated and untreated subjects, respectively, while $s_{\text{treatment}}^{2}$ and $s_{\text{control}}^{2}$ denote the sample variance of the covariate in treated and untreated subjects, respectively.

For dichotomous variables, the ASD is defined as

$$ASD=\frac{p_{\text{treatment}}\text{-}p_{\text{control}}}{\sqrt{\frac{p_{\text{treatment}}\left(1-p_{\text{treatment}}\right)+p_{\text{control}}\left(1-p_{\text{control}}\right)}{2}}}$$

where $p_{\text{treatment}}$ and $p{}_{\text{control}}$ denote the prevalence or mean of the dichotomous variable in treated and untreated subjects, respectively. An ASD of < 0.20 indicates good balance for that particular variable. The baseline characteristics and ASD after PS matching without trimming were described in **Table S1.**

**Sensitivity analyses**

We conducted sensitivity analysis by calculating the SHR using dataset without trimming of extreme data and showed similar results (**Table S2**) indicating a strong robustness of results. In addition, sensitivity analyses with dataset using expanded exclusion criteria were also conducted and observed similar results (**Table S10**).

**Fecal DNA extraction**

Fecal DNA was extracted from the cohort of 157 appendectomy cases (mean age 47 years; 85 males, 72 females) and 157 normal controls (mean age 50 years; 73 males, 84 females) using the QIAamp DNA Stool Mini kit (Qiagen) according to the manufacturer’s instruction.

**Taxonomic profiling**

Taxonomic assignment of high-quality metagenomics reads was performed using exact k-mer based on classification algorithm, by mapping each k-mer in a read to the lowest common ancestor (LCA) of all reference genome as implemented in Kraken2 pipeline using the default settings (1). A more accurate sequence abundance at genus or species levels was re-estimated using Bracken (Bayesian Re-estimation of Abundance after Classification with KrakEN)(2).

**Diversity analysis**

To assess the richness and evenness of the microbial community in appendectomy and healthy control groups, alpha diversity was measured with Shannon and Simpson indices for all the genera classified by Kraken2 in each sample, and the *P*-value was estimated using two-side Wilcoxon test (n = 314). The community dissimilarity between two groups was evaluated by performing Principal Coordinate Analysis (PCoA) analysis based on Bray-Curtis distance. To minimize the error from sequencing, species/genera accounting for at least 1% population in each sample were used only for diversity analysis. The vegan R package (version 2.5.6) was used for the calculation of alpha and beta diversities.

**Co-occurrence network analysis**

To unravel the relationship among differentially abundant species, co-occurrence network based on the metagenomics data was established. The species correlations in the appendectomy and control groups were calculated based on the relative abundance profiles of each species using Spearman’s rank coefficient (rho). The species-species correlation coefficients below -0.2 or above 0.6 were selected for visualization. Due to the intricate relationship among the bacteria, only stronger correlations (abs (rho) appendectomy – abs (rho) normal control > 0.1, rho > 0.6 or rho < -0.2) in the appendectomy group and stronger correlations (abs (rho) normal control – abs (rho) appendectomy > 0.1, rho > 0.6 or rho < -0.2) in normal control group were plotted. To further evaluate the influence of age on the species correlation network, subsampling based on the age (＜50 and ≥ 50 years) was performed and the correlation network was constructed for each subgroup. The Cytoscape (version 3.1.1) was used for the construction of correlation networks.

**Functional profiling**

Functional profiling was carried out using HUMAnN2 across all samples and summarized as MetaCys pathway and KEGG Ontology (KO) gene families(3). The differential pathways were singled out by HUMAnN2 submodule ‘human2_associate’ with default parameters. The KEGG modules under “Amino acid metabolism” and “microbial metabolism” categories were presented with listed KO genes that were analyzed by Mann-Whitney U test with false-discovery rate corrected *P* value (q < 0.1).

**Quantification of bacterial virulence factors**

To uncover the potential effects of intestinal bacteria on the colorectal tumorigenesis, the representative bacteria virulence genes *pks* was identified and quantified using Salmon (v0.14.1) program against a range of viriants of these virulence genes from bacteria. For each sample S, the abundance represented by FPKM of gene were calculated as follows: FPKM = Xi/Li, where Xi is copy number of gene can be detected in the sample, Li is the length of gene i(4).

**Validation of the abundance of candidate bacteria by qPCR**

Abundances of candidate bacteria were estimated in stool samples using SYBRGreen quantitative PCR (qPCR). Primer sequences were designed manually and then tested using Primer Express v3.0 (Applied Biosystems, Foster City, CA) for determination of Tm, GC content and possible secondary structures. Primers were synthesized by Invitrogen (Carlsbad, CA) (**Table S11)**. The abundance of the microbial markers was calculated as a relative unit normalized to the total bacteria of that sample using the 2^−ΔCt^ method (where ΔCt=the average Ct value of each target - the average Ct value of total bacteria)(5).

**Serum LPS Quantification**

The serum LPS level was measured with an ELISA kit (Cloud – Clone Crop., Ltd.,

Wuhan, China). All testing procedures were performed according to the manufacturer’s

instructions.

**Western blot**

The total proteins were isolated from mice colorectum tissue and protein concentration were determined using a BCA assay kit (Thermo Scientific); 10 μg of protein from each sample were separated on 10% SDS–PAGE and then transferred onto PVDF membranes. Blots were incubated with the primary antibodies for ZO-1 tight junction protein (Abcam, #ab276131), E-Cadherin (Abcam, #ab231303), Occludin (Abcam, #ab216327) and GapDH (Proteintech, #60004-1-Ig) overnight at 4 °C and secondary antibodies for 2 hours at room temperature, respectively. Band intensities were determined using ImageJ (National Institutes of Health).

**Immunohistochemistry staining**

Ki-67 （Proteintech, #27309-1-AP）, CD45 (Proteintech, #20103-1-AP), and E-cadherin (Proteintech, #20874-1-AP)staining were performed on paraffin embedded mice colon tumor sections from appendectomy and control mice with normal intestinal microflora. Images were captured using Case Viewer The cell proliferation index was determined by counting the proportion of cells that stained positive for Ki-67 and the expression of E-cadherin in tumor tissue using ImageJ software. Five random microscopic fields were analyzed for each sample.

**RNA sequencing and analysis**

Total RNA was extracted from mice colon tumor tissues. The generation and sequencing of cDNA libraries were performed on NovaSeq 6000 platform to generate 150bp paired-end reads. Clean RNA-seq reads were mapped to Mouse transcriptome (mm10, GRCm38) and quantified in gene-level using Salmon (6) with GENCODE (vM22) gene annotations. Gene set variation analysis (GSVA) were performed on the hallmark pathways, GO biological process and canonical pathways as described in the MSigDB (7). Then we used limma package to detect differentially expressed pathways between groups with t-value. Raw RNA-seq data from this study are available at the Sequence Read Archive (SRA), BioProject ID: PRJNA906334.

**Statistical analysis**

Propensity score (PS) matching was used to analyze and adjust the distribution balance of covariates between the two groups. The balances of covariates between groups were assessed using absolute standardized difference (ASD). For each individual in longitudinal cohort study, we calculated person-years of follow-up until the date of CRC diagnosis, death, or April 1st, 2020. Because of the competition of death for the outcome of interest, we used competing risk model to estimate and calculate the sub-distribution hazard ratio (SHR) and 95% confidence interval (CI) for CRC development after adjusting for confounding variables (age, gender and comorbidities). The epidemiological analyses on subgroup categorized based on age (≤50 years and > 50 years) and gender were also performed. Furthermore, we also analyzed the CRC risk in detailed age subgroup (age ≤50, 50-60, 60-70 and >70 years). We established curve of SHRs-time to assess the time trend of CRC risk by calculating SHRs for CRC incidence at different follow-up years after appendectomy. Sensitivity analyses were conducted by (1) calculating SHRs for cancer incidence using the dataset without trimming of extreme data; and (2) excluding individuals who were diagnosed with cancer within 3 or 5 years after the baseline. *P*-value < 0.05 (two-sided) was considered to be statistically significant. All epidemiological statistical analyses were performed using R Project for Statistical Computing software (version 4.0.2).

In metagenomics sequencing analysis, the difference in microbial community composition was evaluated by pairwise PERMANOVA tests with false discovery rate (FDR) adjustments based on bray distance. LEfSe (Linear discriminant analysis Effect Size) algorithm was used to determine the differentially abundant species and significantly enriched/depleted pathways in the appendectomy group by pairwise comparisons with the control. A corrected *P* < 0.05, by FDR, was considered to be significantly different. The KO genes significantly enriched in appendectomy was identified by comparing to control using Mann-Whitely *U* tests with FDR corrected *P* < 0.1.

**References**

1.Wood DE, Lu J, Langmead B. Improved metagenomic analysis with Kraken 2. Genome Biol 2019;20:257.

2.Lu J, Breitwieser FP, Thielen P, Salzberg SL. Bracken: estimating species abundance in metagenomics data. PeerJ Computer Science 2017;3:e104.

3.Franzosa EA, McIver LJ, Rahnavard G, Thompson LR, Schirmer M, Weingart G, et al. Species-level functional profiling of metagenomes and metatranscriptomes. Nat Methods 2018;15:962-968.

4.Patro R, Duggal G, Love MI, Irizarry RA, Kingsford C. Salmon provides fast and bias-aware quantification of transcript expression. Nat Methods 2017;14:417-419.

5.Wong SH, Kwong T, Chow TC, Luk A, Dai R, Nakatsu G, et al. Quantitation of faecal Fusobacterium improves faecal immunochemical test in detecting advanced colorectal neoplasia. Gut 2017;66:1441-1448.

6.Patro R, Duggal G, Love MI, Irizarry RA, Kingsford C. Salmon provides fast and bias-aware quantification of transcript expression. Nat Methods 2017;14:417-419.

7. Subramanian A, Tamayo P, Mootha VK. Gene set enrichment analysis: a knowledge-based approach for interpreting genome-wide expression profiles. Proc Natl Acad Sci U S A

. 2005 Oct 25;102(43):15545-50.
