# Supplementary material for: Altered gut microbiome composition by appendectomy contributes to colorectal cancer
Source: Oncogene. 2022 Dec 20;42(7):530–40. doi: 10.1038/s41388-022-02569-3 (PMC9918431; doi:10.1038/s41388-022-02569-3)

Supplementary Figure 1. Overview of study design. The study was comprised of an epidemiological study on Cohort 1 (n=416,379) and fecal shotgun metagenomics sequencing study on Cohort 2 (n=513). The flowchart for the epidemiological study as well as the criteria for subject selection on Cohort 1 was illustrated in **(A)**. The Cohort 2 (n=513) was recruited for fecal shotgun metagenomics sequencing study and was filtered based on criteria as illustrated in **(B)**, resulting in a total of 314 samples with age and gender-matched appendectomy and control groups at 1:1 ratio (157 *vs* 157). CRC, colorectal cancer; IBD, inflammatory bowel diseases; OR, odds ratio.

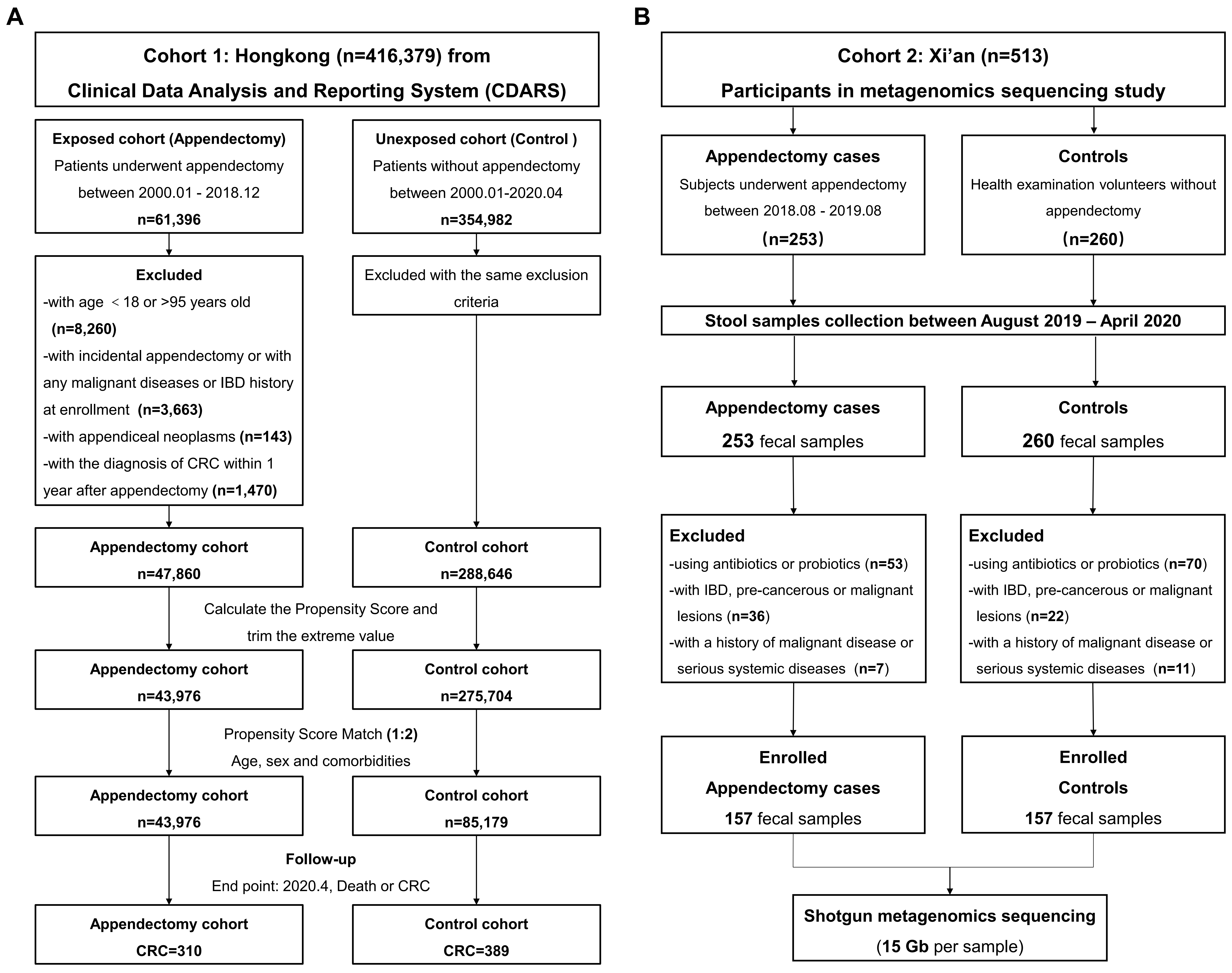

Supplement: Supplementary file 2 — Supplementary Figure 1 [file 41388_2022_2569_MOESM2_ESM.pdf]
