# Supplementary material for: Altered gut microbiome composition by appendectomy contributes to colorectal cancer
Source: Oncogene. 2022 Dec 20;42(7):530–40. doi: 10.1038/s41388-022-02569-3 (PMC9918431; doi:10.1038/s41388-022-02569-3)

Supplementary Figure 2. The subgroup analyses of cumulative incidence of CRC in appendectomy cases and controls classified by gender **(A)** and **(B)**, age **(C)** and **(D)**, and tumor locations **(E)**, **(F)**, **(G)** and **(H)**. Proximal colon cancer included the tumor located in cecum, ascending colon, hepatic flexure and transverse colon. Distal colon cancer included the tumors located in splenic flexure, descending colon and sigmoid colon.

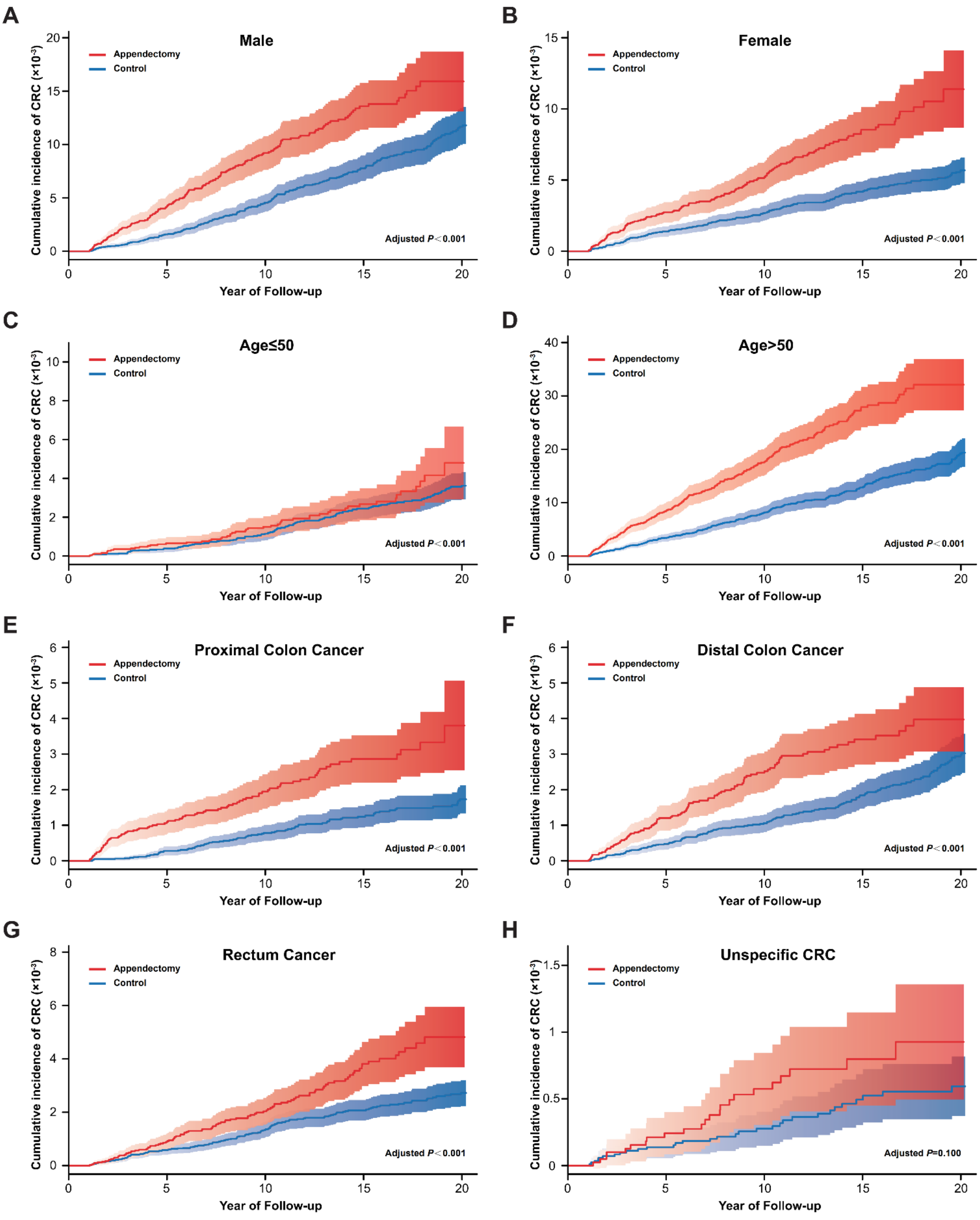

Supplement: Supplementary file 3 — Supplementary Figure 2 [file 41388_2022_2569_MOESM3_ESM.pdf]
