# Supplementary material for: Altered gut microbiome composition by appendectomy contributes to colorectal cancer
Source: Oncogene. 2022 Dec 20;42(7):530–40. doi: 10.1038/s41388-022-02569-3 (PMC9918431; doi:10.1038/s41388-022-02569-3)

Supplementary Figure 3. The temporal trends of the adjusted SHR in appendectomy cases over the 20 follow-up years stratified by gender **(A)** and age **(B)**.

A

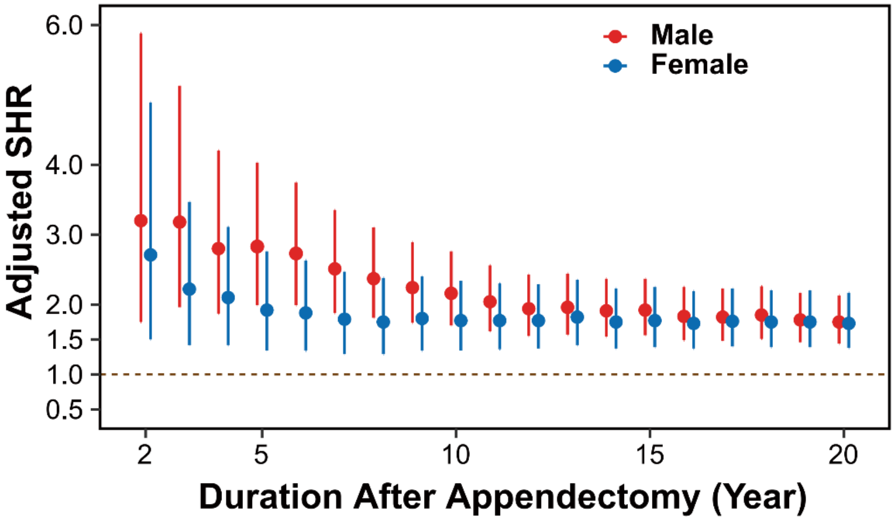

B

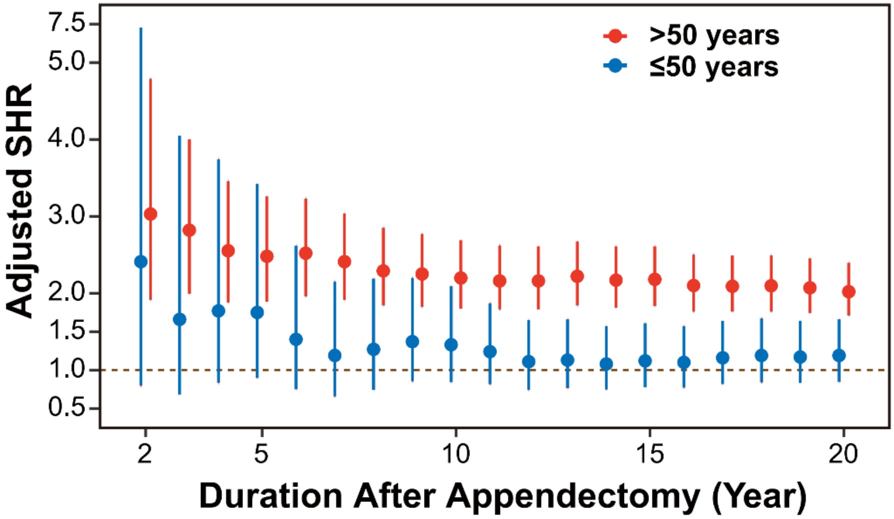

Supplement: Supplementary file 4 — Supplementary Figure 3 [file 41388_2022_2569_MOESM4_ESM.pdf]
