# Supplementary material for: Altered gut microbiome composition by appendectomy contributes to colorectal cancer
Source: Oncogene. 2022 Dec 20;42(7):530–40. doi: 10.1038/s41388-022-02569-3 (PMC9918431; doi:10.1038/s41388-022-02569-3)

Supplementary Figure 5. Relative abundance of selected appendectomy-enriched bacterial species in all subjects

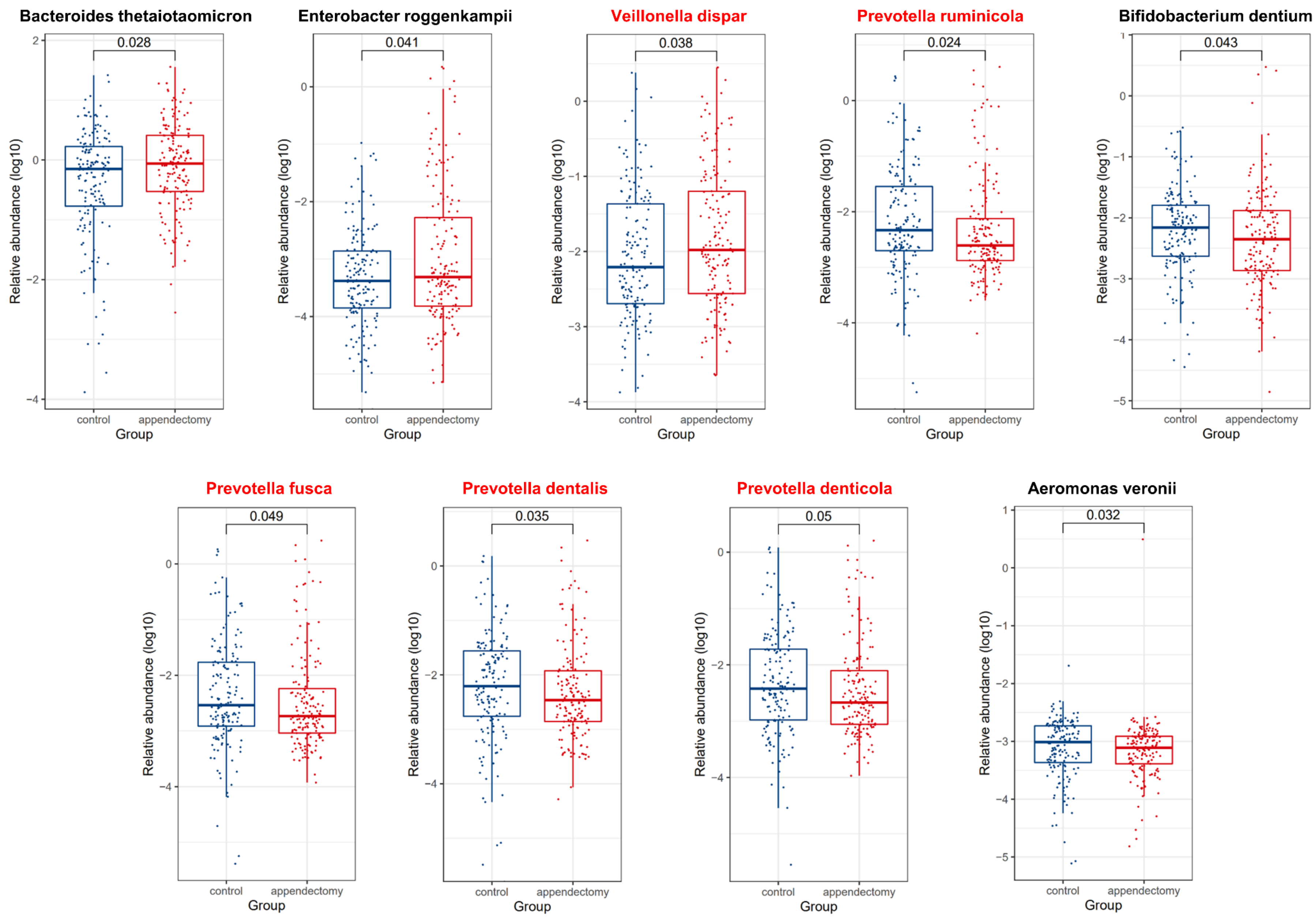

Supplement: Supplementary file 6 — Supplementary Figure 5 [file 41388_2022_2569_MOESM6_ESM.pdf]
