# Supplementary material for: Altered gut microbiome composition by appendectomy contributes to colorectal cancer
Source: Oncogene. 2022 Dec 20;42(7):530–40. doi: 10.1038/s41388-022-02569-3 (PMC9918431; doi:10.1038/s41388-022-02569-3)

**Supplementary Figure 7. (A)** The changes of relative abundance of selected bacteria (*B. vulgatus*, *B. fragilis*, *B. sp SC05B48* and *C. aerofaciens*) during different time span after appendectomy; **(B)** The analysis of specific bacterial genetic element associated with the development of CRC**.**

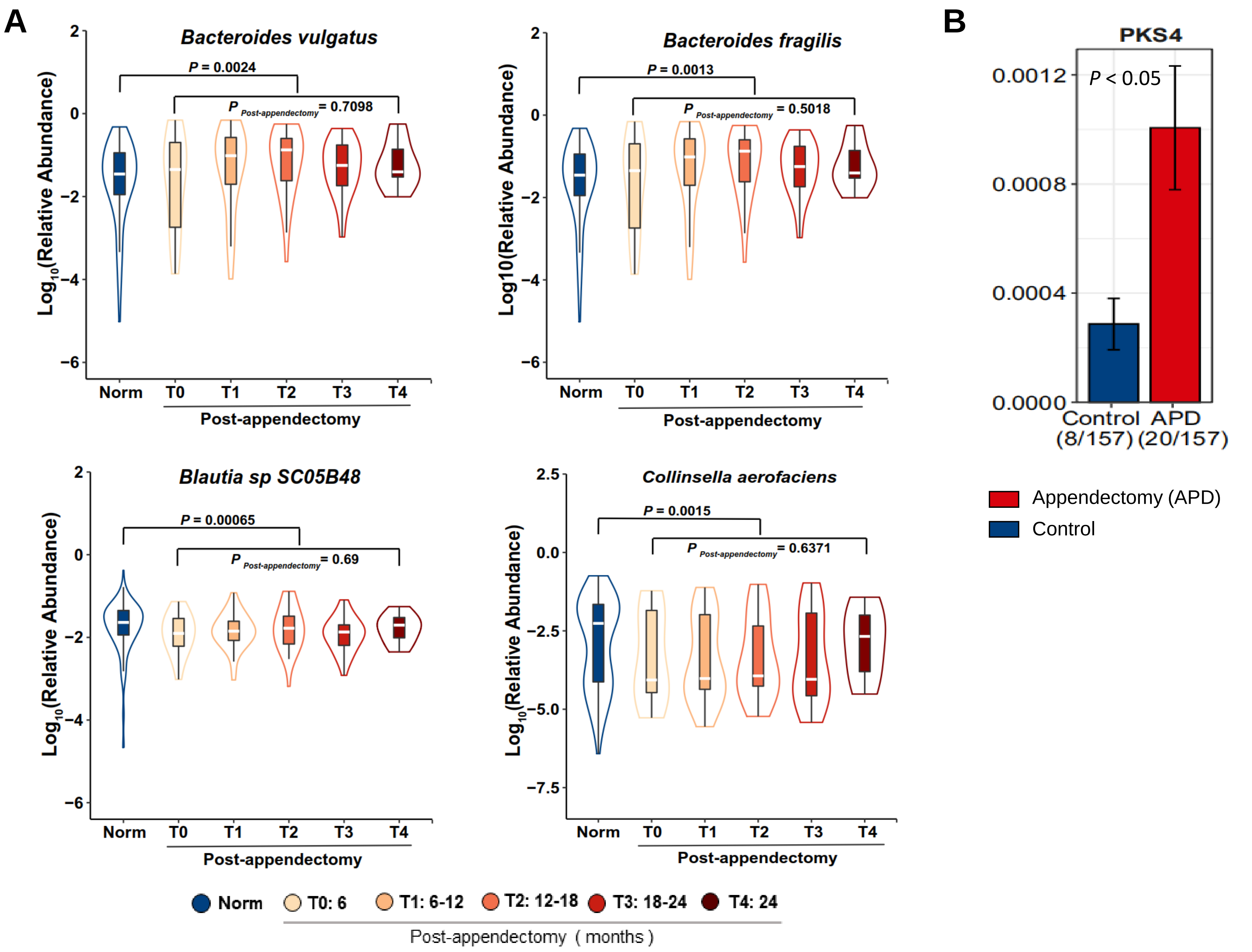

Supplement: Supplementary file 8 — Supplementary Figure 7 [file 41388_2022_2569_MOESM8_ESM.pdf]
