# Supplementary material for: Altered gut microbiome composition by appendectomy contributes to colorectal cancer
Source: Oncogene. 2022 Dec 20;42(7):530–40. doi: 10.1038/s41388-022-02569-3 (PMC9918431; doi:10.1038/s41388-022-02569-3)

**Supplementary Figure 8.** Quantitative PCR melt curves of the microbial marker *Bacteroides fragilis* **(A)**, *Bacteroides vulgatus* **(B)**, *Veillonella dispar* **(C)**, *Enterococcus hirae* **(D)**, *Lachnospiraceae bacterium Choco86* **(E)**, *Blautia sp. SC05B48* **(F)**, respectively.

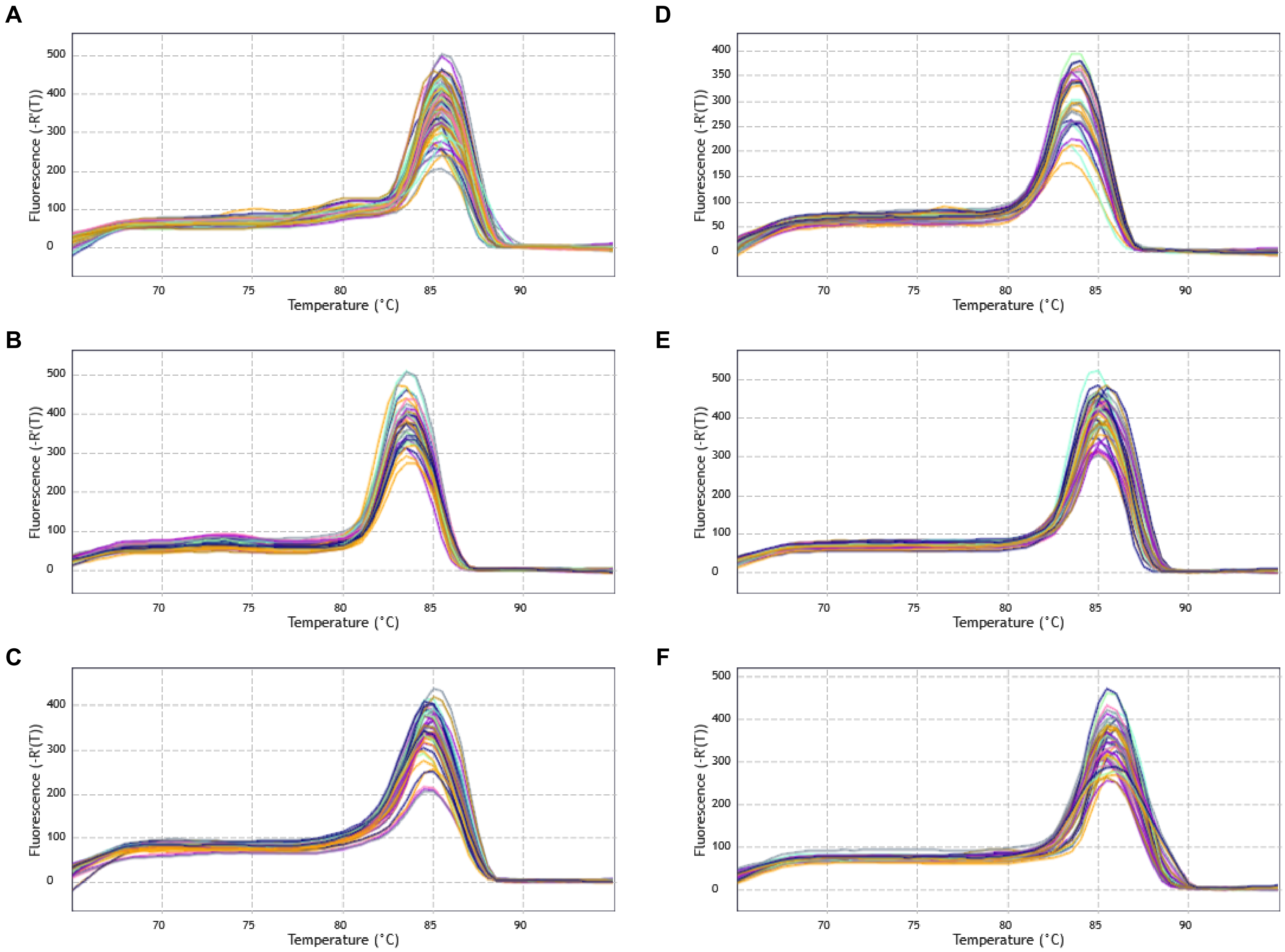

Supplement: Supplementary file 9 — Supplementary Figure 8 [file 41388_2022_2569_MOESM9_ESM.pdf]
