# Supplementary material for: Altered gut microbiome composition by appendectomy contributes to colorectal cancer
Source: Oncogene. 2022 Dec 20;42(7):530–40. doi: 10.1038/s41388-022-02569-3 (PMC9918431; doi:10.1038/s41388-022-02569-3)

Supplementary Figure 9 (A) Body weight between female and male mice in appendectomy and control groups; **(B)** Differences in pathway activities scored by GSVA between appendectomy and control groups in mice by RNA sequencing.

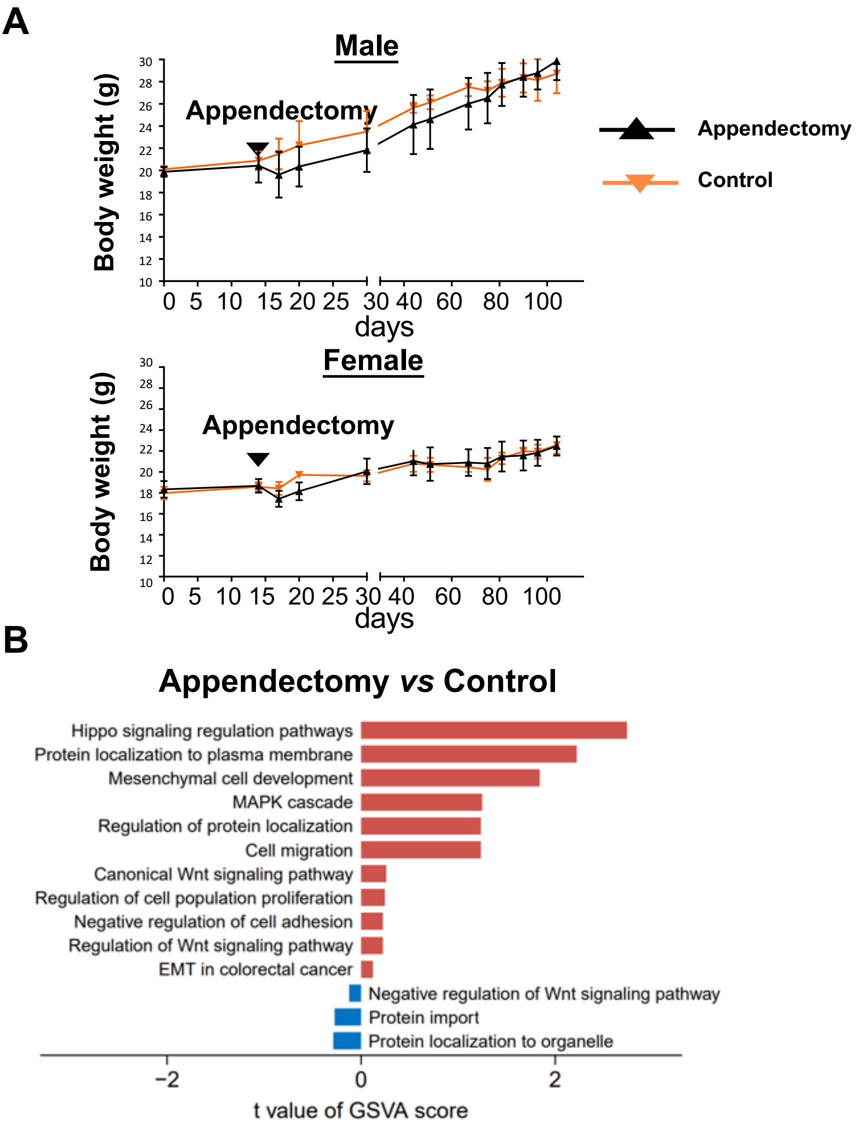

Supplement: Supplementary file 10 — Supplementary Figure 9 [file 41388_2022_2569_MOESM10_ESM.pdf]
