# Supplementary material for: Altered gut microbiome composition by appendectomy contributes to colorectal cancer
Source: Oncogene. 2022 Dec 20;42(7):530–40. doi: 10.1038/s41388-022-02569-3 (PMC9918431; doi:10.1038/s41388-022-02569-3)

Supplementary Figure 10. The intestinal bacteria dysbiosis caused by appendectomy play a key role in CRC tumorigenesis. (A) Schematic diagram of a cocktail of broad-spectrum antibiotics treated AOM/DSS-induced CRC mouse model. (**B**) The level of total bacteria in stool samples of mice was determined by qPCR. **(C)** Body weight in male and female mice treated with appendectomy or control; **(D)** Representative images of colon and evaluation of colon tumor load from control and appendectomy groups in male and female mice.

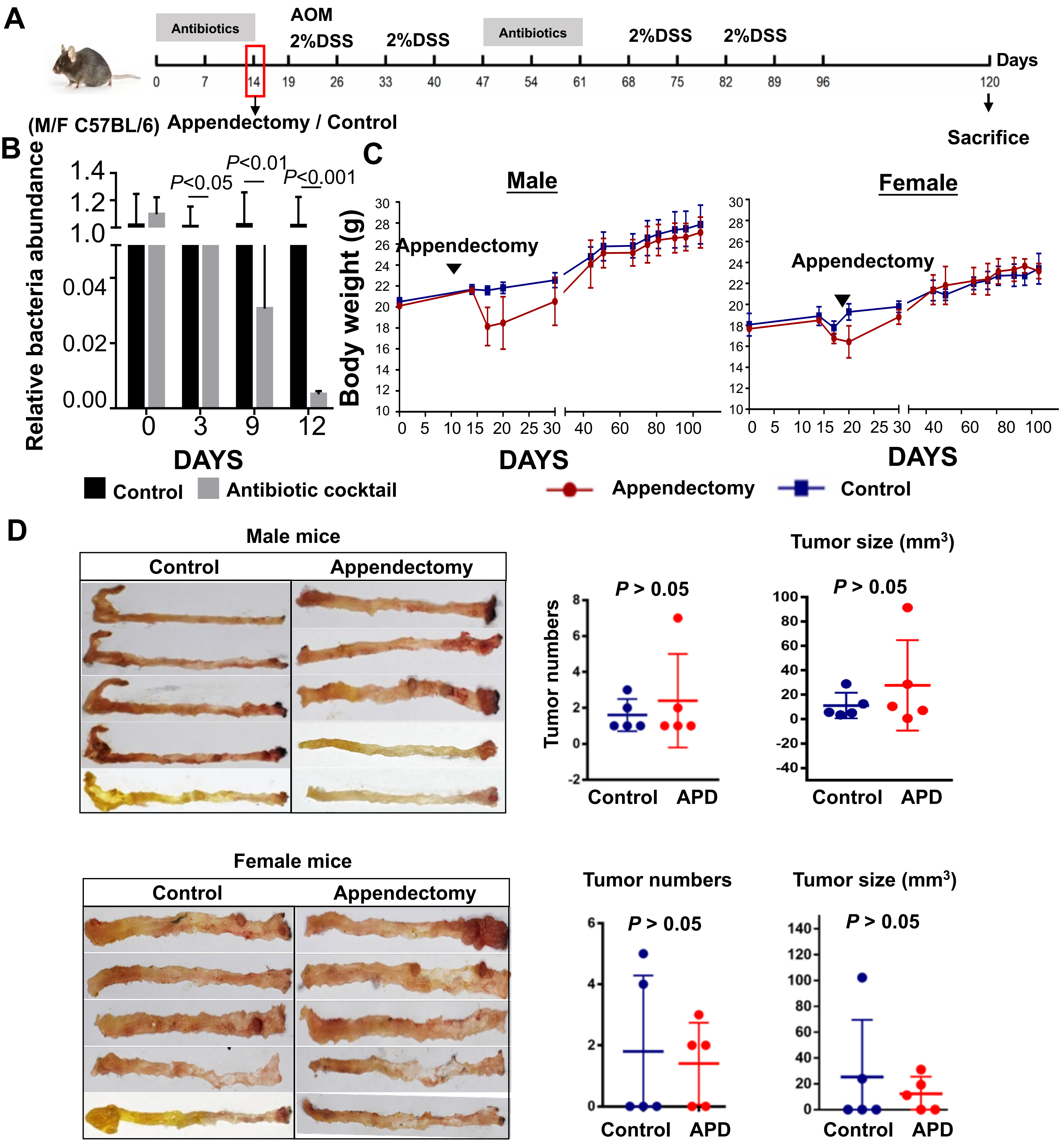

Supplement: Supplementary file 11 — Supplementary Figure 10 [file 41388_2022_2569_MOESM11_ESM.pdf]
