# Supplementary material for: Altered gut microbiome composition by appendectomy contributes to colorectal cancer
Source: Oncogene. 2022 Dec 20;42(7):530–40. doi: 10.1038/s41388-022-02569-3 (PMC9918431; doi:10.1038/s41388-022-02569-3)
Supplement: Supplementary file 12 — Supplementary Table 1-11 [file 41388_2022_2569_MOESM12_ESM.pdf]

Supplementary Table 1: Baseline features of study data set before and after PS matching with and without trimming.

	All (n=336,506)	Before PS matching		ASD*	After PS matching with trimming**		ASD*	After PS matching without trimming**		ASD*
		Appendectomy (n=47,860)	Control (n=288,645)		Appendectomy (n=43,976)	Control (n=85,179)		Appendectomy (n=47,860)	Control (n=89,766)	
Age, years, median (IQR)	57 (38-75)	42 (29-56)	61 (40-76)	0.746	44 (32-58)	44 (33-58)	0.032	42 (29-56)	43(31-57)	0.072
Male sex, No. (%)	151,832 (45.12)	23,913 (49.96)	127,919 (44.32)	0.113	20,042 (45.57)	37,346 (43.84)	0.035	23,913 (49.96)	41,887 (46.66)	0.066
DM, No. (%)	36,714 (10.91)	1,283 (2.68)	35,431 (12.27)	0.371	1,271 (2.89)	2,541 (2.98)	0.006	1,283 (2.68)	2,552 (2.84)	0.010
Hypertension, No. (%)	38,316 (11.39)	1,145 (2.39)	37,171 (12.88)	0.403	1,130 (2.57)	2,233 (2.62)	0.003	1,145 (2.39)	2,264 (2.52)	0.008
Hyperlipidemia, No. (%)	10,114 (3.01)	316 (0.66)	9,798 (3.39)	0.195	310 (0.70)	608 (0.71)	0.001	316 (0.66)	603 (0.67)	0.001
CHF, No. (%)	16,012 (4.76)	233 (0.49)	15,779 (5.47)	0.296	223 (0.51)	424 (0.50)	0.001	233 (0.49)	427 (0.48)	0.002
RD, No. (%)	1,107 (0.33)	50 (0.10)	1,057 (0.37)	0.054	50 (0.11)	94 (0.11)	0.001	50 (0.10)	97 (0.11)	0.001
Dementia, No. (%)	4,898 (1.46)	54 (0.11)	4,844 (1.68)	0.167	53 (0.12)	91 (0.11)	0.004	54 (0.11)	97 (0.11)	0.001
Mild liver disease, No. (%)	1,548 (0.46)	26 (0.05)	1,522 (0.53)	0.088	24 (0.05)	51 (0.06)	0.002	26 (0.05)	51 (0.06)	0.001
Severe liver disease, No. (%)	947 (0.28)	7 (0.01)	940 (0.33)	0.076	7 (0.02)	19 (0.02)	0.005	7 (0.01)	24 (0.03)	0.008
MI, No. (%)	6,061 (1.80)	97 (0.20)	5,964 (2.07)	0.177	90 (0.20)	178 (0.21)	0.001	97 (0.20)	174 (0.19)	0.002
Paraplegia, No. (%)	2,639 (0.78)	51 (0.11)	2,588 (0.90)	0.112	48 (0.11)	74 (0.09)	0.007	51 (0.11)	74 (0.08)	0.008
Peptic ulcer disease, No. (%)	4,960 (1.47)	216 (0.45)	4,744 (1.64)	0.117	213 (0.48)	375 (0.44)	0.007	216 (0.45)	357 (0.40)	0.008
CPD, No. (%)	16,222 (4.82)	484 (1.01)	15,738 (5.45)	0.253	480 (1.09)	931 (1.09)	<0.001	484 (1.01)	906 (1.01)	<0.001
Renal disease, No. (%)	12,521 (3.72)	181 (0.38)	12,340 (4.28)	0.261	173 (0.39)	301 (0.35)	0.007	181 (0.38)	333 (0.37)	0.001
Stroke, No. (%)	19,921 (5.92)	412 (0.86)	19,509 (6.76)	0.312	402 (0.91)	732 (0.86)	0.006	412 (0.86)	782 (0.87)	0.001

Continuous variables were expressed as median (IQR).

Categorical variables were expressed as number (%).

*Variables with an ASD >0.20 is considered to be imbalanced.

**PS matching was performed after trimming of the extreme PS strata (2.5th and 97.5th percentiles). Non-appendectomy individuals were matched to appendectomy patients on PS within a calliper width of 0.02. All variables were included in the model for PS estimation.

ASD: absolute standardized difference; CHF: Congestive heart failure; CPD: Chronic pulmonary disease; DM: diabetes mellitus; MI: Myocardial infarction; PS: propensity score; PVD: Peripheral vascular disease; RD: Rheumatologic disease.

Supplementary Table 2. Sensitivity analysis on assessing the risk of CRC after PS matching with and without trimming.

	PS adjustment with trimming						PS adjustment without trimming					
	Appendectomy		Control		Adjusted SHR* (95% CI)	*P*	Appendectomy		Control		Adjusted SHR* (95% CI)	*P*
	NO. of event	Incidence rate** (95% CI)	NO. of event	Incidence rate** (95% CI)			NO. of event	Incidence rate** (95% CI)	NO. of event	Incidence rate** (95% CI)		
CRC	310	73.1 (65.0-81.2）	388	39.7 (35.8-43.7)	1.730 (1.490-2.010)	<0.001	310	66.4 (59-73.8)	389	37.7 (34-41.4)	1.710 (1.480-1.990)	<0.001
Age≤50	53	18.1 (13.3-23.0)	113	16.3 (13.3-19.4)	1.190 (0.848-1.660)	0.320	53	15.8 (11.6-20.1)	114	15.3 (12.5-18.1)	1.180 (0.842-1.640)	0.340
Age>50	257	194.9 (171.1-218.7)	275	96.3 (84.9-107.7)	2.020 (1.710-2.396)	<0.001	257	194.8 (171.0-218.6)	275	96.3 (84.9-107.7)	2.023 (1.710-2.395)	<0.001
Female	135	57.6 (47.9-67.4）	171	29.4 (25.0-33.8)	1.730 (1.380-2.170)	<0.001	135	57.6 (47.9-67.3)	171	29.4 (25-33.8)	1.710 (1.360-2.170)	<0.001
Male	175	92.1 (78.5-105.8）	217	54.8 (47.5-62.1)	1.750 (1.440-2.130)	<0.001	175	75.3 (64.1-86.4)	218	48.4 (42-54.8)	1.750 (1.450-2.130)	<0.001
Proximal colon	85	20.1 (15.8-24.4）	83	8.5 (6.7-10.4)	2.210 (1.640-2.990)	<0.001	85	18.3 (14.4-22.2)	84	8.2 (6.4-9.9)	2.160 (1.600-2.920)	<0.001
Distal colon	101	23.9 (19.2-28.6）	136	14 (11.6-16.3)	1.670 (1.290-2.150)	<0.001	101	21.7 (17.5-25.9)	136	13.2 (11-15.4)	1.650 (1.290-2.130)	<0.001
Rectum	101	23.9 (19.2-28.5）	138	14.2 (11.8-16.5)	1.570 (1.210-2.020)	<0.001	101	21.7 (17.5-25.9)	138	13.4 (11.2-15.6)	1.550 (1.200-2.000)	0.001
Unspecific	23	5.4 (3.2-7.7）	31	3.2 (2.1-4.3)	1.560 (0.914-2.650)	0.100	23	4.9 (2.9-7.0)	31	3.0 (2.0-4.1)	1.560 (0.915-2.650)	0.098

*Adjusted by Age, Gender, Diabetes mellitus, Hypertension, Hyperlipidemia, diabetes mellitus, hypertension, hyperlipidemia, congestive heart failure, rheumatic disease, dementia, liver disease (divided into mild, moderate, or severe liver disease according to the severity of portal hypertension), myocardial infarction, paraplegia, peptic ulcer, chronic pulmonary disease, chronic kidney disease, and stroke.

**Per 10000 person-years.

CRC: colorectal cancer; CI: confidence interval; PS: propensity score; SHR: subdistribution hazard ratio.

Supplementary Table 3. Demographic and clinical characteristics of all included samples in metagenomics sequencing study

	**Appendectomy (n=157)**	**Controls (n=157)**	***p***
**Age, year**			0.309
⩽50	85 (54.14%)	76 (48.41%)	
＞50	72 (45.86%)	81 (51.59%)	
**Sex, n**			0.176
Female	72 (45.86%)	84 (53.50%)	
Male	85 (54.14%)	73 (46.50%)	
**BMI, kg/m^2**	23.10 ± 2.63	22.72 ± 2.73	0.214
**Smoke status, n (%)**			0.324
No	122 (77.71%)	129 (82.17%)	
Current	35 (22.29%)	28 (17.83%)	
**Alcohol status, n (%)**			0.682
No	143 (91.08%)	145 (92.36%)	
Yes	14 (8.92%)	12 (7.64%)	
**Diabetes mellitus, n (%)**			0.791
No	149 (94.90%)	150 (95.54%)	
Yes	8 (5.10%)	7 (4.46%)	
**Family history of cancer, n (%)**			0.355
No	151 (96.2%)	147 (93.6%)	
Other cancer	5 (3.2%)	6 (3.8%)	
Colorectal cancer	1 (0.6%)	4 (2.5%)	
**History of appendicitis*, n (%)**			0.104
No	146 (93.0%)	151 (96.2%)	
Yes	3 (1.9%)	0 (0.0%)	
Suspected	8 (5.1%)	6 (3.8%)	

*History of appendicitis was defined as any imaging evidence of chronic appendicitis before appendectomy (for appendectomy group) or fecal collection (for controls).

Supplementary Table 4. Sequencing reads by shotgun metagenomics sequencing

Sample	raw pair1	raw pair2	trimmed pair1	trimmed pair2	trimmed orphan1	trimmed orphan2	decontaminated Homo_sapiens pair1	decontaminated Homo_sapiens pair2	decontaminated Homo_sapiens orphan1	decontaminated Homo_sapiens orphan2	final pair1	final pair2	final orphan1	final orphan2
A100	56093741	56093741	40459197	40459197	6928745	3917448	40438683	40438683	6924952	3915305	40438683	40438683	6924952	3915305
A101	51301931	51301931	29843134	29843134	11089271	3352163	29840876	29840876	11088285	3351868	29840876	29840876	11088285	3351868
A102	52128697	52128697	33486951	33486951	8957180	3532548	33486467	33486467	8957023	3532523	33486467	33486467	8957023	3532523
A103	52358420	52358420	30258798	30258798	11882952	3209754	30257673	30257673	11882471	3209608	30257673	30257673	11882471	3209608
A104	50333619	50333619	32515656	32515656	8669926	3423529	32515346	32515346	8669839	3423484	32515346	32515346	8669839	3423484
A010	53222675	53222675	33149529	33149529	9419192	3667263	33147301	33147301	9418549	3667036	33147301	33147301	9418549	3667036
A011	52885727	52885727	29854950	29854950	11544728	3492122	29852104	29852104	11543725	3491903	29852104	29852104	11543725	3491903
A012	50555180	50555180	34274062	34274062	6698238	3612575	34268701	34268701	6697222	3611940	34268701	34268701	6697222	3611940
A013	54973750	54973750	36009152	36009152	9561532	3369053	35986659	35986659	9554585	3366419	35986659	35986659	9554585	3366419
A014	58651958	58651958	39025308	39025308	9202455	3958655	39022942	39022942	9201515	3958122	39022942	39022942	9201515	3958122
A015	56157112	56157112	36977017	36977017	9374243	3574356	36976689	36976689	9374143	3574329	36976689	36976689	9374143	3574329
A016	57054180	57054180	35975783	35975783	11139636	3226624	35975437	35975437	11139502	3226595	35975437	35975437	11139502	3226595
A017	52018393	52018393	37631332	37631332	6279657	3825124	37630699	37630699	6279606	3825084	37630699	37630699	6279606	3825084
A018	54573835	54573835	37891412	37891412	7345876	3812579	37868448	37868448	7341665	3810306	37868448	37868448	7341665	3810306
A019	55969648	55969648	39449487	39449487	7685776	3925853	39446734	39446734	7685208	3925561	39446734	39446734	7685208	3925561
A001	56289099	56289099	39026954	39026954	7590085	3938225	39025965	39025965	7590092	3938331	39025965	39025965	7590092	3938331
A020	56643542	56643542	38174927	38174927	8400131	3880720	38163529	38163529	8397491	3879535	38163529	38163529	8397491	3879535
A021	53932584	53932584	36608567	36608567	7991014	3572996	36603843	36603843	7981648	3563495	36603843	36603843	7981648	3563495
A022	55442592	55442592	36443041	36443041	9497634	3506993	36431346	36431346	9500080	3510274	36431346	36431346	9500080	3510274
A023	55522835	55522835	34193948	34193948	10454096	3869563	34184855	34184855	10451145	3868447	34184855	34184855	10451145	3868447
A024	55715787	55715787	37396723	37396723	7513038	4228427	37395896	37395896	7512837	4228320	37395896	37395896	7512837	4228320
A025	52815099	52815099	34552264	34552264	7603733	4173724	34551880	34551880	7603661	4173682	34551880	34551880	7603661	4173682
A026	55078485	55078485	30675602	30675602	13215094	3799846	30623804	30623804	13188502	3792093	30623804	30623804	13188502	3792093
A027	53073596	53073596	33434649	33434649	8916406	3938107	33324783	33324783	8883645	3925139	33324783	33324783	8883645	3925139
A028	51554522	51554522	32697222	32697222	8966221	3482895	32691176	32691176	8964602	3482189	32691176	32691176	8964602	3482189
A029	50347432	50347432	32144140	32144140	8115124	3801909	22704978	22704978	5344846	2539757	22704978	22704978	5344846	2539757
A002	57191890	57191890	38818791	38818791	9097736	3424852	38812572	38812572	9095327	3423587	38812572	38812572	9095327	3423587
A030	55777549	55777549	36512408	36512408	8462753	3969597	36325977	36325977	8407202	3943437	36325977	36325977	8407202	3943437
A031	52357950	52357950	34318819	34318819	8223108	3959720	34237466	34237466	8198198	3947863	34237466	34237466	8198198	3947863
A032	50932351	50932351	32016417	32016417	8653209	3776394	32013456	32013456	8652385	3776038	32013456	32013456	8652385	3776038
A033	52572391	52572391	33763997	33763997	7264942	4784061	33763006	33763006	7264658	4783848	33763006	33763006	7264658	4783848
A034	51593319	51593319	29592389	29592389	11478432	3221112	29572042	29572042	11469838	3218787	29572042	29572042	11469838	3218787
A036	57421432	57421432	39460373	39460373	7798606	4235956	39458341	39458341	7798069	4235673	39458341	39458341	7798069	4235673
A037	54728394	54728394	37130674	37130674	8158922	3569565	36224225	36224225	7955087	3476201	36224225	36224225	7955087	3476201
A038	54084864	54084864	37389726	37389726	7532072	3710179	37230280	37230280	7495828	3693101	37230280	37230280	7495828	3693101
A039	55213712	55213712	31954198	31954198	11374367	3945635	31952628	31952628	11373804	3945449	31952628	31952628	11373804	3945449
A003	56354851	56354851	36598177	36598177	10098745	3307569	36553450	36553450	10087311	3303742	36553450	36553450	10087311	3303742
A040	53935314	53935314	34864091	34864091	8389579	4058382	34810992	34810992	8375405	4051178	34810992	34810992	8375405	4051178
A041	54999541	54999541	34457399	34457399	9621109	4021951	34456474	34456474	9620810	4021801	34456474	34456474	9620810	4021801
A042	56443328	56443328	35479192	35479192	9892181	3863456	35478475	35478475	9891967	3863367	35478475	35478475	9891967	3863367
A043	50981231	50981231	32588068	32588068	8037428	3919973	32568332	32568332	8033053	3917775	32568332	32568332	8033053	3917775
A044	53366817	53366817	34503119	34503119	9218563	3764853	34502491	34502491	9218395	3764818	34502491	34502491	9218395	3764818
A045	51402047	51402047	31182146	31182146	9662858	3698365	31171772	31171772	9659647	3697218	31171772	31171772	9659647	3697218
A046	53570327	53570327	33946945	33946945	9896146	3432287	33943408	33943408	9894878	3431911	33943408	33943408	9894878	3431911
A047	50021731	50021731	33540952	33540952	7567051	3413470	33540515	33540515	7566968	3413446	33540515	33540515	7566968	3413446
A048	53249928	53249928	34409674	34409674	9220113	3457495	34408445	34408445	9219782	3457422	34408445	34408445	9219782	3457422

A049	50671465	50671465	32704438	32704438	7704793	4220931	32702362	32702362	7704429	4220711	32702362	32702362	7704429	4220711
A004	56249470	56249470	36781948	36781948	9538713	3579145	36778220	36778220	9537836	3578831	36778220	36778220	9537836	3578831
A050	51025434	51025434	29963698	29963698	11199996	3282623	29961285	29961285	11199026	3282278	29961285	29961285	11199026	3282278
A051	54784853	54784853	33589288	33589288	11396403	3000850	33588995	33588995	11396290	3000835	33588995	33588995	11396290	3000835
A052	54130563	54130563	34424793	34424793	10256324	3435172	34424317	34424317	10256190	3435114	34424317	34424317	10256190	3435114
A053	51206549	51206549	35768609	35768609	6177136	4003070	35766408	35766408	6176718	4002803	35766408	35766408	6176718	4002803
A054	54325847	54325847	35354257	35354257	8330013	4157215	35352583	35352583	8329506	4156980	35352583	35352583	8329506	4156980
A055	51805222	51805222	32842563	32842563	9607214	3149334	32840431	32840431	9606641	3149168	32840431	32840431	9606641	3149168
A056	58176140	58176140	38035207	38035207	9908282	3575169	38033415	38033415	9907714	3574898	38033415	38033415	9907714	3574898
A057	54156772	54156772	36451991	36451991	8199781	3703380	36413264	36413264	8189969	3698897	36413264	36413264	8189969	3698897
A058	54850424	54850424	37936757	37936757	7754916	3522421	37926376	37926376	7752939	3521455	37926376	37926376	7752939	3521455
A059	55766900	55766900	38900272	38900272	7852272	4170032	38899505	38899505	7852019	4169933	38899505	38899505	7852019	4169933
A005	56921431	56921431	36156643	36156643	10373627	3726267	32102076	32102076	9105433	3265025	32102076	32102076	9105433	3265025
A060	56029970	56029970	37335727	37335727	9340796	3327119	36833611	36833611	9193244	3275681	36833611	36833611	9193244	3275681
A061	52702163	52702163	36628847	36628847	7309923	3232049	36587028	36587028	7300366	3228383	36587028	36587028	7300366	3228383
A062	54104837	54104837	37767298	37767298	7152096	3746123	37765314	37765314	7151725	3745911	37765314	37765314	7151725	3745911
A063	50633215	50633215	33280626	33280626	8174448	3446092	33264809	33264809	8170152	3444213	33264809	33264809	8170152	3444213
A064	52348534	52348534	37359722	37359722	5885452	3805656	37354590	37354590	5884336	3804969	37354590	37354590	5884336	3804969
A065	51081048	51081048	35439868	35439868	7111020	3083452	35438943	35438943	7110740	3083253	35438943	35438943	7110740	3083253
A066	55896200	55896200	38413515	38413515	7946908	3850614	38236983	38236983	7904380	3831958	38236983	38236983	7904380	3831958
A067	54196169	54196169	36434119	36434119	7751855	4270633	36428672	36428672	7750332	4269712	36428672	36428672	7750332	4269712
A068	55594904	55594904	36587535	36587535	10347843	3601745	36562924	36562924	10341022	3599890	36562924	36562924	10341022	3599890
A069	54109105	54109105	38270947	38270947	7999520	3687522	38270683	38270683	7999457	3687477	38270683	38270683	7999457	3687477
A006	54751940	54751940	32502749	32502749	11190828	3691979	32501647	32501647	11190288	3691781	32501647	32501647	11190288	3691781
A070	53830865	53830865	33267071	33267071	10710537	3395970	33264640	33264640	10709816	3395715	33264640	33264640	10709816	3395715
A072	57575659	57575659	39618634	39618634	9025855	3982704	39611969	39611969	9024107	3981961	39611969	39611969	9024107	3981961
A073	59045975	59045975	40640571	40640571	8675957	4153688	40626393	40626393	8671548	4150393	40626393	40626393	8671548	4150393
A075	56121345	56121345	37140017	37140017	8845401	3939518	37112323	37112323	8835491	3933782	37112323	37112323	8835491	3933782
A076	50685064	50685064	35835986	35835986	6423275	3451441	35833916	35833916	6422827	3451071	35833916	35833916	6422827	3451071
A077	54336131	54336131	37659159	37659159	7597207	3524490	37117300	37117300	7489159	3477151	37117300	37117300	7489159	3477151
A078	72730759	72730759	48247873	48247873	12037416	4209049	48229334	48229334	12032896	4207635	48229334	48229334	12032896	4207635
A079	51576697	51576697	35992762	35992762	6864963	3417744	35988557	35988557	6863926	3416918	35988557	35988557	6863926	3416918
A007	54116250	54116250	34238091	34238091	9124217	3749476	34120010	34120010	9096066	3737691	34120010	34120010	9096066	3737691
A080	59617716	59617716	41159456	41159456	8474199	3626973	41157547	41157547	8473783	3626861	41157547	41157547	8473783	3626861
A081	55486847	55486847	29581750	29581750	12066852	4198875	29579046	29579046	12065748	4198468	29579046	29579046	12065748	4198468
A082	54814713	54814713	36051008	36051008	9689936	3198725	33944824	33944824	9155729	2992671	33944824	33944824	9155729	2992671
A083	54021765	54021765	36669180	36669180	8210972	3252042	36661933	36661933	8209447	3251297	36661933	36661933	8209447	3251297
A084	56373274	56373274	36990956	36990956	9488397	3312668	36989576	36989576	9488092	3312587	36989576	36989576	9488092	3312587
A085	53556610	53556610	35810021	35810021	8515331	3392329	35807512	35807512	8514794	3392102	35807512	35807512	8514794	3392102
A086	52334664	52334664	29415907	29415907	9405614	4743609	29197249	29197249	9324667	4701988	29197249	29197249	9324667	4701988
A087	51155787	51155787	30405102	30405102	11280973	3082707	30404758	30404758	11280856	3082673	30404758	30404758	11280856	3082673
A088	54093789	54093789	36959050	36959050	7621833	3860234	36956742	36956742	7621297	3860019	36956742	36956742	7621297	3860019
A089	50392003	50392003	34371330	34371330	8559633	3287893	34365272	34365272	8557835	3287115	34365272	34365272	8557835	3287115
A008	50869189	50869189	32635986	32635986	8366986	3468203	32635661	32635661	8366895	3468150	32635661	32635661	8366895	3468150
A091	52833066	52833066	34128486	34128486	9313801	3354391	34090671	34090671	9300903	3350101	34090671	34090671	9300903	3350101
A092	58107375	58107375	40088110	40088110	9445326	3783771	40087754	40087754	9445225	3783732	40087754	40087754	9445225	3783732
A093	57005660	57005660	38382209	38382209	9838590	3529575	38375141	38375141	9836993	3529029	38375141	38375141	9836993	3529029
A094	51175474	51175474	34711406	34711406	7224688	3804344	34699996	34699996	7221638	3802897	34699996	34699996	7221638	3802897
A095	54560335	54560335	37062958	37062958	7687599	4045025	37062308	37062308	7687414	4044952	37062308	37062308	7687414	4044952
A096	54150633	54150633	31081684	31081684	13927228	2904883	31080385	31080385	13925336	2903774	31080385	31080385	13925336	2903774
A097	55971088	55971088	33228604	33228604	11647675	3431258	33222087	33222087	11644728	3430505	33222087	33222087	11644728	3430505

A098	53560738	53560738	34561008	34561008	8853758	3535075	34559101	34559101	8853378	3535000	34559101	34559101	8853378	3535000
A099	50387431	50387431	30154923	30154923	9132579	4049633	30154027	30154027	9132245	4049467	30154027	30154027	9132245	4049467
A009	54308338	54308338	33632501	33632501	10069806	3593962	33625906	33625906	10067617	3593109	33625906	33625906	10067617	3593109
B012	53073541	53073541	28580562	28580562	9791499	5018873	28547779	28547779	9796189	5025705	28547779	28547779	9796189	5025705
B013	55426524	55426524	31270014	31270014	9778873	4818442	31269341	31269341	9778619	4818319	31269341	31269341	9778619	4818319
B014	54773272	54773272	39267042	39267042	7213967	4048579	39265926	39265926	7213745	4048476	39265926	39265926	7213745	4048476
B015	54415987	54415987	35678028	35678028	9305649	3306174	35672691	35672691	9304153	3305618	35672691	35672691	9304153	3305618
B016	52067120	52067120	36314037	36314037	7075353	3337256	36189530	36189530	7051760	3325903	36189530	36189530	7051760	3325903
B017	53849240	53849240	38374597	38374597	6476151	3791836	38370669	38370669	6475377	3791399	38370669	38370669	6475377	3791399
B018	54208448	54208448	37553882	37553882	7418043	3685760	37550552	37550552	7417307	3685283	37550552	37550552	7417307	3685283
B019	54227080	54227080	36948937	36948937	9582453	2973179	36948293	36948293	9582252	2973127	36948293	36948293	9582252	2973127
B001	56551558	56551558	37617719	37617719	8271512	3916414	37542640	37542640	8249648	3906830	37542640	37542640	8249648	3906830
B020	53029875	53029875	36322482	36322482	8002370	3261264	36322184	36322184	8002305	3261221	36322184	36322184	8002305	3261221
B021	51222463	51222463	36952202	36952202	5951406	3259809	36537425	36537425	5872782	3219388	36537425	36537425	5872782	3219388
B022	53737290	53737290	37876888	37876888	6783715	3635112	37471962	37471962	6706971	3596310	37471962	37471962	6706971	3596310
B023	56093289	56093289	40006657	40006657	6639781	3811404	39996064	39996064	6638420	3810712	39996064	39996064	6638420	3810712
B024	50750407	50750407	35627896	35627896	5775116	3994833	35620954	35620954	5773907	3993976	35620954	35620954	5773907	3993976
B025	54619171	54619171	34471768	34471768	11881738	3310311	34470377	34470377	11881433	3310373	34470377	34470377	11881433	3310373
B026	56126244	56126244	39706532	39706532	7742395	4159866	39705166	39705166	7742115	4159691	39705166	39705166	7742115	4159691
B027	54786230	54786230	38240708	38240708	7987941	4027714	38234989	38234989	7986769	4027159	38234989	38234989	7986769	4027159
B028	51877168	51877168	26932518	26932518	11373171	4106414	26916785	26916785	11365676	4103468	26916785	26916785	11365676	4103468
B029	53890383	53890383	38249300	38249300	7311386	3830070	38238033	38238033	7309035	3828767	38238033	38238033	7309035	3828767
B002	55179517	55179517	31589172	31589172	12547812	3347408	31573086	31573086	12540532	3345409	31573086	31573086	12540532	3345409
B030	53570719	53570719	30612460	30612460	9469839	4671340	30606356	30606356	9467509	4670174	30606356	30606356	9467509	4670174
B031	56293754	56293754	31584073	31584073	9991601	5016832	31423993	31423993	9935357	4986906	31423993	31423993	9935357	4986906
B032	51898103	51898103	37295005	37295005	6821465	3559386	37293397	37293397	6821114	3559199	37293397	37293397	6821114	3559199
B034	54733103	54733103	31135458	31135458	9532373	4987994	31134906	31134906	9532117	4987777	31134906	31134906	9532117	4987777
B035	51414319	51414319	30812423	30812423	9915872	3697488	30811732	30811732	9915699	3697415	30811732	30811732	9915699	3697415
B036	56281404	56281404	34821459	34821459	10403247	3701542	34821018	34821018	10403120	3701497	34821018	34821018	10403120	3701497
B037	51101825	51101825	31032781	31032781	9598353	3626844	30905424	30905424	9560310	3612657	30905424	30905424	9560310	3612657
B038	53351679	53351679	34456538	34456538	10373962	3462285	34445893	34445893	10370101	3461183	34445893	34445893	10370101	3461183
B039	54596136	54596136	35394068	35394068	8601867	4042421	35318573	35318573	8585083	4035015	35318573	35318573	8585083	4035015
B003	52683152	52683152	33794162	33794162	8502332	3760625	33765369	33765369	8494575	3757290	33765369	33765369	8494575	3757290
B040	50270666	50270666	30444423	30444423	9706743	3441767	30442757	30442757	9706128	3441579	30442757	30442757	9706128	3441579
B041	53172278	53172278	34057761	34057761	8638994	3872346	34054279	34054279	8638078	3872013	34054279	34054279	8638078	3872013
B042	57305311	57305311	35329913	35329913	10389294	4016237	35328068	35328068	10388705	4015994	35328068	35328068	10388705	4015994
B043	53082183	53082183	35783801	35783801	9187273	3393954	35783014	35783014	9187056	3393867	35783014	35783014	9187056	3393867
B044	53963359	53963359	32160235	32160235	10762677	3791317	32155308	32155308	10760825	3790779	32155308	32155308	10760825	3790779
B045	54243318	54243318	38374944	38374944	7279415	3926930	33503393	33503393	6220445	3345773	33503393	33503393	6220445	3345773
B046	53600782	53600782	37549955	37549955	6527867	4618898	37544003	37544003	6525700	4617358	37544003	37544003	6525700	4617358
B047	56207775	56207775	39963572	39963572	7824855	3814173	39962000	39962000	7824468	3813983	39962000	39962000	7824468	3813983
B048	54388067	54388067	38646229	38646229	6881742	3972385	38643821	38643821	6881283	3972122	38643821	38643821	6881283	3972122
B049	52820059	52820059	28042363	28042363	10919738	4231730	28040437	28040437	10918757	4231355	28040437	28040437	10918757	4231355
B004	54177620	54177620	36837710	36837710	6851012	4230324	36822649	36822649	6848209	4228916	36822649	36822649	6848209	4228916
B050	54896721	54896721	36843337	36843337	10194912	2821920	36842337	36842337	10194369	2821674	36842337	36842337	10194369	2821674
B051	53020309	53020309	35074200	35074200	8217944	3728966	35060687	35060687	8214100	3727223	35060687	35060687	8214100	3727223
B052	51732165	51732165	35024404	35024404	7104443	3973729	35023728	35023728	7104267	3973634	35023728	35023728	7104267	3973634
B053	50547711	50547711	33745595	33745595	7547597	3751592	33745268	33745268	7547481	3751547	33745268	33745268	7547481	3751547
B054	53764682	53764682	37407521	37407521	6527343	4281058	37406787	37406787	6527197	4280970	37406787	37406787	6527197	4280970
B056	50555155	50555155	33292624	33292624	8073654	3409365	33292411	33292411	8073577	3409326	33292411	33292411	8073577	3409326
B057	56285755	56285755	35419814	35419814	10741662	3281111	35418979	35418979	10739541	3279552	35418979	35418979	10739541	3279552

B058	50685959	50685959	33033402	33033402	8221851	3665035	33032952	33032952	8221760	3665020	33032952	33032952	8221760	3665020
B059	52215267	52215267	35272014	35272014	7103950	4134529	35266190	35266190	7099379	4130417	35266190	35266190	7099379	4130417
B005	50958682	50958682	34784426	34784426	8677684	3296687	34783996	34783996	8677573	3296626	34783996	34783996	8677573	3296626
B060	53628488	53628488	35403834	35403834	8430392	3804705	35403371	35403371	8430241	3804578	35403371	35403371	8430241	3804578
B061	55633263	55633263	35743499	35743499	10266351	3261749	35700089	35700089	10251951	3256638	35700089	35700089	10251951	3256638
B062	53107066	53107066	36184429	36184429	7509903	3711209	36184078	36184078	7509834	3711207	36184078	36184078	7509834	3711207
B006	54413285	54413285	31090862	31090862	10097710	4559142	30647557	30647557	9940401	4483664	30647557	30647557	9940401	4483664
B008	55871122	55871122	40122541	40122541	7044213	4075792	40118892	40118892	7044603	4076388	40118892	40118892	7044603	4076388
B009	56880947	56880947	40705341	40705341	7936100	3824984	40703908	40703908	7935737	3824795	40703908	40703908	7935737	3824795
C100	55663808	55663808	27267583	27267583	13522170	4060137	27260068	27260068	13517797	4058788	27260068	27260068	13517797	4058788
C101	56082481	56082481	28145871	28145871	13079655	4343359	28145192	28145192	13078916	4342851	28145192	28145192	13078916	4342851
C010	50495765	50495765	25479032	25479032	12056841	3717029	25463991	25463991	12050391	3714722	25463991	25463991	12050391	3714722
C011	55471169	55471169	27418717	27418717	12716972	4174327	27418317	27418317	12716794	4174258	27418317	27418317	12716794	4174258
C012	53197209	53197209	36484477	36484477	8531672	3496421	36420957	36420957	8511656	3486778	36420957	36420957	8511656	3486778
C013	54103309	54103309	36628219	36628219	7621347	3912423	31683665	31683665	6392542	3294185	31683665	31683665	6392542	3294185
C014	53274603	53274603	26136609	26136609	12739336	3894829	26136364	26136364	12739214	3894796	26136364	26136364	12739214	3894796
C015	50516190	50516190	26071502	26071502	10682901	4105641	25975647	25975647	10634812	4086189	25975647	25975647	10634812	4086189
C016	53571648	53571648	28259918	28259918	10649037	4634629	28227754	28227754	10634712	4628398	28227754	28227754	10634712	4628398
C017	51774435	51774435	27878563	27878563	10056723	4530606	27878072	27878072	10056576	4530528	27878072	27878072	10056576	4530528
C018	57723390	57723390	40219760	40219760	7115031	4542452	38284950	38284950	6738053	4316341	38284950	38284950	6738053	4316341
C019	55500889	55500889	29165110	29165110	11879849	4663117	29161176	29161176	11878223	4662500	29161176	29161176	11878223	4662500
C001	54746061	54746061	35102392	35102392	8612836	4223114	35014717	35014717	8591897	4214474	35014717	35014717	8591897	4214474
C020	57458081	57458081	30025509	30025509	11692106	5096894	29579969	29579969	11488624	5009207	29579969	29579969	11488624	5009207
C021	50994534	50994534	31358145	31358145	9207705	3698524	31356846	31356846	9207280	3698331	31356846	31356846	9207280	3698331
C022	52705558	52705558	33778949	33778949	9591075	3279121	33778005	33778005	9590739	3279006	33778005	33778005	9590739	3279006
C023	53943435	53943435	35129287	35129287	9006789	3924291	35102537	35102537	8999915	3921436	35102537	35102537	8999915	3921436
C024	50086011	50086011	33278234	33278234	7587905	3669761	33278016	33278016	7587846	3669728	33278016	33278016	7587846	3669728
C025	66665380	66665380	47711001	47711001	9204063	4530530	47708943	47708943	9203593	4530318	47708943	47708943	9203593	4530318
C026	54653958	54653958	35745576	35745576	7995264	4223601	35733642	35733642	7992812	4222328	35733642	35733642	7992812	4222328
C027	59492969	59492969	36294889	36294889	10913938	4294548	36293918	36293918	10913685	4294456	36293918	36293918	10913685	4294456
C028	59251031	59251031	31879293	31879293	13092969	4318805	31878475	31878475	13092581	4318683	31878475	31878475	13092581	4318683
C029	52211490	52211490	28525913	28525913	10041415	4710896	28522739	28522739	10040418	4710506	28522739	28522739	10040418	4710506
C002	52460197	52460197	29200907	29200907	9913492	4432655	29173808	29173808	9902782	4427798	29173808	29173808	9902782	4427798
C030	53234221	53234221	34119045	34119045	8598508	3803372	34072877	34072877	8586884	3798375	34072877	34072877	8586884	3798375
C031	63953748	63953748	33797709	33797709	13954212	4941248	33797517	33797517	13954096	4941212	33797517	33797517	13954096	4941212
C032	58600776	58600776	28406115	28406115	14784422	4157032	28405680	28405680	14784140	4156958	28405680	28405680	14784140	4156958
C033	58912087	58912087	32359170	32359170	11475190	4932955	32358797	32358797	11474995	4932878	32358797	32358797	11474995	4932878
C034	54305951	54305951	27440707	27440707	12120207	4232539	27440254	27440254	12119981	4232449	27440254	27440254	12119981	4232449
C035	58860458	58860458	31500759	31500759	11023422	5502304	30915391	30915391	10783874	5381009	30915391	30915391	10783874	5381009
C036	53705026	53705026	27727966	27727966	11160223	4577726	27722864	27722864	11157711	4576646	27722864	27722864	11157711	4576646
C037	56922426	56922426	29622095	29622095	11780013	4892715	29540517	29540517	11742285	4876992	29540517	29540517	11742285	4876992
C038	51955662	51955662	33861409	33861409	8443632	3539873	33859110	33859110	8442887	3539664	33859110	33859110	8442887	3539664
C003	53269437	53269437	32943461	32943461	9141840	4168800	32938357	32938357	9140064	4168212	32938357	32938357	9140064	4168212
C040	54545494	54545494	36473757	36473757	8089085	3941443	36471367	36471367	8085888	3939073	36471367	36471367	8085888	3939073
C041	53431287	53431287	33998735	33998735	9954436	3154335	33644840	33644840	9834810	3115715	33644840	33644840	9834810	3115715
C042	52592451	52592451	37198611	37198611	6784882	3354221	37196767	37196767	6784624	3354131	37196767	37196767	6784624	3354131
C043	53302917	53302917	34114297	34114297	9791631	3130864	34106243	34106243	9789235	3129807	34106243	34106243	9789235	3129807
C044	53060967	53060967	36936338	36936338	7079356	3745631	36926972	36926972	7077595	3744629	36926972	36926972	7077595	3744629
C045	52076280	52076280	36453399	36453399	6853051	3572587	36451938	36451938	6852825	3572456	36451938	36451938	6852825	3572456
C046	51854805	51854805	35909012	35909012	7307802	3295089	35900133	35900133	7305901	3294320	35900133	35900133	7305901	3294320
C047	53813213	53813213	38981104	38981104	6131417	3427635	38722461	38722461	6082603	3400725	38722461	38722461	6082603	3400725

C048	51590165	51590165	33387129	33387129	7972721	3562765	33384289	33384289	7972041	3562374	33384289	33384289	7972041	3562374
C049	51287108	51287108	34536014	34536014	8005817	3171805	34414305	34414305	7975163	3160563	34414305	34414305	7975163	3160563
C004	52058545	52058545	35860483	35860483	6941508	3611337	35644724	35644724	6891260	3586331	35644724	35644724	6891260	3586331
C050	51483785	51483785	34058436	34058436	8442936	3250524	34052688	34052688	8439945	3248623	34052688	34052688	8439945	3248623
C051	52663000	52663000	34735878	34735878	9233377	2954967	34227519	34227519	9074779	2904834	34227519	34227519	9074779	2904834
C052	62108669	62108669	31463563	31463563	14871216	4531959	31462140	31462140	14870314	4531676	31462140	31462140	14870314	4531676
C053	53390830	53390830	34491948	34491948	8112727	3763092	34490612	34490612	8111786	3762456	34490612	34490612	8111786	3762456
C054	53119364	53119364	35903475	35903475	7665477	3537991	35903207	35903207	7665400	3537937	35903207	35903207	7665400	3537937
C056	54877883	54877883	35078087	35078087	10220176	3273869	35069545	35069545	10217434	3272612	35069545	35069545	10217434	3272612
C057	54204642	54204642	37682275	37682275	7730134	3404480	37682060	37682060	7730105	3404447	37682060	37682060	7730105	3404447
C058	52626262	52626262	34825180	34825180	8327188	3707464	34819926	34819926	8325668	3706686	34819926	34819926	8325668	3706686
C059	51567016	51567016	32455908	32455908	10369400	2837016	32453208	32453208	10369419	2837441	32453208	32453208	10369419	2837441
C005	50945389	50945389	31418264	31418264	9159196	3738398	31301742	31301742	9120252	3722485	31301742	31301742	9120252	3722485
C060	53781986	53781986	36466784	36466784	7932740	3524870	36392898	36392898	7918871	3518272	36392898	36392898	7918871	3518272
C061	52149900	52149900	35053924	35053924	7726185	3827248	35052177	35052177	7725937	3827199	35052177	35052177	7725937	3827199
C062	52202503	52202503	34074061	34074061	8793260	3475371	34069997	34069997	8792058	3474918	34069997	34069997	8792058	3474918
C063	52259184	52259184	34327618	34327618	8529830	3480486	34326588	34326588	8529537	3480361	34326588	34326588	8529537	3480361
C064	51771031	51771031	34138769	34138769	8229934	3503355	34135876	34135876	8229255	3503047	34135876	34135876	8229255	3503047
C065	51706597	51706597	34900907	34900907	7212334	3832904	34891684	34891684	7210211	3831792	34891684	34891684	7210211	3831792
C066	54178703	54178703	36131936	36131936	8214555	3954874	36131337	36131337	8214334	3954789	36131337	36131337	8214334	3954789
C067	53848904	53848904	33782219	33782219	9843619	3874864	33778982	33778982	9842438	3874329	33778982	33778982	9842438	3874329
C068	54036783	54036783	36386127	36386127	7480158	4379003	36385315	36385315	7479994	4378921	36385315	36385315	7479994	4378921
C069	54560342	54560342	36881537	36881537	7950486	3916353	36876484	36876484	7949278	3915830	36876484	36876484	7949278	3915830
C006	65460845	65460845	40962394	40962394	10867383	5227559	40937261	40937261	10860386	5224535	40937261	40937261	10860386	5224535
C070	51109250	51109250	36368024	36368024	5636147	3976310	36364843	36364843	5635032	3975285	36364843	36364843	5635032	3975285
C071	51835280	51835280	28120462	28120462	9485596	4819670	28120156	28120156	9485465	4819611	28120156	28120156	9485465	4819611
C072	56232571	56232571	30432093	30432093	10140458	5216069	30321934	30321934	10096738	5193258	30321934	30321934	10096738	5193258
C073	53221974	53221974	37863803	37863803	7408906	3673350	36667605	36667605	7110823	3527409	36667605	36667605	7110823	3527409
C074	52550088	52550088	26743897	26743897	11593580	4173212	26743585	26743585	11593333	4173114	26743585	26743585	11593333	4173114
C075	53395507	53395507	28960649	28960649	9543938	5079119	28960120	28960120	9543711	5079001	28960120	28960120	9543711	5079001
C076	53773642	53773642	28287271	28287271	11041207	4445711	28264320	28264320	11031693	4441523	28264320	28264320	11031693	4441523
C077	56081033	56081033	28811929	28811929	12594095	4006450	28784767	28784767	12579746	4001633	28784767	28784767	12579746	4001633
C078	52599241	52599241	27054540	27054540	10968146	4500575	27052023	27052023	10967070	4500124	27052023	27052023	10967070	4500124
C079	56096458	56096458	29287609	29287609	11687234	4721238	29285934	29285934	11686440	4720905	29285934	29285934	11686440	4720905
C007	56929850	56929850	35684189	35684189	9896691	4077647	35547771	35547771	9861230	4066041	35547771	35547771	9861230	4066041
C080	54173049	54173049	27388919	27388919	12393175	4084333	27387982	27387982	12392578	4084053	27387982	27387982	12392578	4084053
C082	51318325	51318325	33980176	33980176	8026435	3529879	33979331	33979331	8026135	3529738	33979331	33979331	8026135	3529738
C083	54667528	54667528	36438675	36438675	10269539	3317916	36435266	36435266	10268238	3317234	36435266	36435266	10268238	3317234
C084	51513749	51513749	30030658	30030658	9075089	4124471	29881489	29881489	9018358	4095879	29881489	29881489	9018358	4095879
C085	50985861	50985861	28266784	28266784	8435675	5332371	28262620	28262620	8434492	5331613	28262620	28262620	8434492	5331613
C086	53169757	53169757	34407299	34407299	8515890	3813682	34405965	34405965	8515601	3813521	34405965	34405965	8515601	3813521
C087	53490896	53490896	32159127	32159127	10371760	3619149	32018878	32018878	10338622	3607534	32018878	32018878	10338622	3607534
C088	49509706	49509706	32078117	32078117	7586853	3756806	32076607	32076607	7586570	3756757	32076607	32076607	7586570	3756757
C089	55397450	55397450	38010121	38010121	9321084	3145286	37926684	37926684	9295384	3136627	37926684	37926684	9295384	3136627
C008	52140080	52140080	36025633	36025633	8304635	3553650	35869303	35869303	8260902	3536090	35869303	35869303	8260902	3536090
C090	49950048	49950048	33382709	33382709	6641212	4068945	33382197	33382197	6641057	4068856	33382197	33382197	6641057	4068856
C091	54454114	54454114	35761014	35761014	7725074	4379931	35755091	35755091	7723592	4379248	35755091	35755091	7723592	4379248
C092	58265074	58265074	27875883	27875883	15458645	3925233	27875502	27875502	15458432	3925147	27875502	27875502	15458432	3925147
C093	50241876	50241876	27592385	27592385	9736386	4232148	27530708	27530708	9713846	4221252	27530708	27530708	9713846	4221252
C094	51678885	51678885	34199000	34199000	7971011	3613422	34153271	34153271	7960461	3608300	34153271	34153271	7960461	3608300
C095	54685295	54685295	28267979	28267979	11573440	4285542	28254690	28254690	11563592	4279698	28254690	28254690	11563592	4279698

C096	58705029	58705029	30268995	30268995	12577962	4703967	30265628	30265628	12576413	4703363	30265628	30265628	12576413	4703363
C097	60039903	60039903	26463494	26463494	16084576	4149108	26461924	26461924	16083602	4148844	26461924	26461924	16083602	4148844
C098	50886876	50886876	26932902	26932902	9845845	4842712	26925062	26925062	9842534	4840996	26925062	26925062	9842534	4840996
C099	56995608	56995608	27946084	27946084	13417337	4355774	27941749	27941749	13415416	4355078	27941749	27941749	13415416	4355078
C009	52178530	52178530	33251391	33251391	8130172	4151802	33190650	33190650	8112132	4142747	33190650	33190650	8112132	4142747
D010	52878490	52878490	35142555	35142555	7556094	3811686	35137182	35137182	7554845	3811161	35137182	35137182	7554845	3811161
D011	54603291	54603291	37361440	37361440	7842354	3615345	37359343	37359343	7841846	3615129	37359343	37359343	7841846	3615129
D012	55731079	55731079	36592629	36592629	8027275	4311493	35506745	35506745	7808534	4196831	35506745	35506745	7808534	4196831
D013	56615736	56615736	36112164	36112164	8240152	4847623	36110687	36110687	8239660	4848035	36110687	36110687	8239660	4848035
D014	52785386	52785386	33348761	33348761	9581318	3299985	33302228	33302228	9564775	3294530	33302228	33302228	9564775	3294530
D015	57054008	57054008	36693585	36693585	9134597	4251117	36645326	36645326	9123168	4246808	36645326	36645326	9123168	4246808
D016	58063610	58063610	36937907	36937907	9797997	4029234	35283237	35283237	9258232	3835569	35283237	35283237	9258232	3835569
D017	52333518	52333518	35764791	35764791	7402302	3832396	35761192	35761192	7401289	3831817	35761192	35761192	7401289	3831817
D018	56481973	56481973	38763989	38763989	7046301	4246397	38761143	38761143	7045811	4246125	38761143	38761143	7045811	4246125
D019	52321935	52321935	34907563	34907563	7551691	4060651	34906520	34906520	7551413	4060534	34906520	34906520	7551413	4060534
D001	57115703	57115703	28888993	28888993	12258441	4892002	28888345	28888345	12258142	4891891	28888345	28888345	12258142	4891891
D020	54425797	54425797	32870184	32870184	10613479	3609613	32868817	32868817	10612970	3609453	32868817	32868817	10612970	3609453
D021	55293463	55293463	36459569	36459569	7986659	4086019	36456573	36456573	7985945	4085691	36456573	36456573	7985945	4085691
D022	57487360	57487360	38408870	38408870	6981332	4824824	38406180	38406180	6980775	4824456	38406180	38406180	6980775	4824456
D023	54426252	54426252	36114687	36114687	8543099	3549010	36113810	36113810	8542820	3548900	36113810	36113810	8542820	3548900
D024	50181234	50181234	23902261	23902261	10867576	4532056	23895186	23895186	10862888	4529328	23895186	23895186	10862888	4529328
D025	53579694	53579694	28245459	28245459	11817419	4318652	28244927	28244927	11817221	4318588	28244927	28244927	11817221	4318588
D026	55669333	55669333	35081618	35081618	10140558	3935744	35079188	35079188	10139726	3935411	35079188	35079188	10139726	3935411
D027	52569857	52569857	27847791	27847791	11102249	4065228	27847482	27847482	11102102	4065180	27847482	27847482	11102102	4065180
D028	51631142	51631142	34119587	34119587	7944742	3716363	34119111	34119111	7944638	3716317	34119111	34119111	7944638	3716317
D029	53022222	53022222	35585491	35585491	7648194	3890826	35490606	35490606	7622927	3878916	35490606	35490606	7622927	3878916
D002	62672809	62672809	32924211	32924211	12842246	5357356	32922701	32922701	12841174	5356565	32922701	32922701	12841174	5356565
D030	59885859	59885859	39969456	39969456	9114703	4529432	39884923	39884923	9098708	4522551	39884923	39884923	9098708	4522551
D031	52214592	52214592	34225220	34225220	8271860	3766695	34221107	34221107	8270889	3766348	34221107	34221107	8270889	3766348
D032	53280263	53280263	35833390	35833390	7618686	4065694	35833078	35833078	7618617	4065645	35833078	35833078	7618617	4065645
D033	53810545	53810545	30061106	30061106	13733063	3147608	30057495	30057495	13731104	3147140	30057495	30057495	13731104	3147140
D035	55576633	55576633	35591561	35591561	9778856	3702313	35581373	35581373	9775882	3701291	35581373	35581373	9775882	3701291
D036	51439715	51439715	28703503	28703503	9586537	4430469	28701994	28701994	9585998	4430214	28701994	28701994	9585998	4430214
D037	52011529	52011529	29529263	29529263	9362683	4489038	29507003	29507003	9354013	4484944	29507003	29507003	9354013	4484944
D038	53340602	53340602	28818875	28818875	11033786	4224508	28369801	28369801	10829920	4141956	28369801	28369801	10829920	4141956
D039	53697728	53697728	27031267	27031267	11516655	4544402	27030966	27030966	11516521	4544354	27030966	27030966	11516521	4544354
D003	59183516	59183516	30535538	30535538	13519191	4506413	30534216	30534216	13518595	4506201	30534216	30534216	13518595	4506201
D040	56825645	56825645	35372300	35372300	11919788	3275554	35362271	35362271	11909823	3267207	35362271	35362271	11909823	3267207
D041	53149267	53149267	29720759	29720759	9726993	4608934	29719726	29719726	9726504	4608633	29719726	29719726	9726504	4608633
D042	57847957	57847957	30422322	30422322	12149490	4556826	30421732	30421732	12149211	4556708	30421732	30421732	12149211	4556708
D043	52810680	52810680	35867324	35867324	7604723	3874759	35860184	35860184	7602953	3873832	35860184	35860184	7602953	3873832
D044	53097715	53097715	29860271	29860271	9281698	4545616	29859802	29859802	9281497	4545575	29859802	29859802	9281497	4545575
D045	62587389	62587389	33116466	33116466	13134906	5018191	33106591	33106591	13129872	5016246	33106591	33106591	13129872	5016246
D046	53119249	53119249	33762970	33762970	8728394	3546775	33104809	33104809	8545525	3478023	33104809	33104809	8545525	3478023
D047	52867032	52867032	34055544	34055544	9183875	3586662	34047696	34047696	9181694	3585596	34047696	34047696	9181694	3585596
D048	52240204	52240204	35993055	35993055	7438268	3232540	35962112	35962112	7431357	3229617	35962112	35962112	7431357	3229617
D049	52136237	52136237	36635209	36635209	6948152	3393492	35647663	35647663	6725075	3285725	35647663	35647663	6725075	3285725
D004	66996674	66996674	36616827	36616827	13460650	5273180	36616492	36616492	13460517	5273145	36616492	36616492	13460517	5273145
D050	53995690	53995690	37699632	37699632	7376836	3357947	37533905	37533905	7342734	3341924	37533905	37533905	7342734	3341924
D051	54015066	54015066	36839560	36839560	7964575	3539855	36773602	36773602	7950078	3533857	36773602	36773602	7950078	3533857
D052	53539142	53539142	36263388	36263388	8206913	3297447	36261687	36261687	8206501	3297322	36261687	36261687	8206501	3297322

D053	50766454	50766454	34903144	34903144	7424424	3094628	34896120	34896120	7423459	3094382	34896120	34896120	7423459	3094382
D054	53706682	53706682	39028417	39028417	6018566	3503546	39027271	39027271	6018393	3503442	39027271	39027271	6018393	3503442
D055	53665499	53665499	37169040	37169040	7125671	3809155	37161528	37161528	7124163	3808475	37161528	37161528	7124163	3808475
D056	52304586	52304586	34425327	34425327	7933201	3841106	34421960	34421960	7932457	3840785	34421960	34421960	7932457	3840785
D057	50085496	50085496	24976454	24976454	11136128	4172297	21450285	21450285	9415028	3477372	21450285	21450285	9415028	3477372
D058	51223098	51223098	24591115	24591115	12843494	3419027	24519244	24519244	12799473	3404625	24519244	24519244	12799473	3404625
D059	63382409	63382409	35228299	35228299	14089387	4938542	35227672	35227672	14089059	4938359	35227672	35227672	14089059	4938359
D005	54343942	54343942	36491591	36491591	7248811	4170177	36258478	36258478	7209287	4151291	36258478	36258478	7209287	4151291
D060	52656441	52656441	25035767	25035767	13073516	4073170	25034441	25034441	13072696	4072946	25034441	25034441	13072696	4072946
D006	54755792	54755792	36421339	36421339	7855054	3932707	36373538	36373538	7843288	3926628	36373538	36373538	7843288	3926628
D007	51570057	51570057	35252598	35252598	6856979	3896882	35252224	35252224	6856867	3896822	35252224	35252224	6856867	3896822
D008	52543695	52543695	32108613	32108613	9902561	3630302	32025333	32025333	9875794	3621013	32025333	32025333	9875794	3621013
D009	53664621	53664621	34165269	34165269	8481240	4177263	34142926	34142926	8474302	4174115	34142926	34142926	8474302	4174115

Note: Appendectomy cases: A and B; Controls: C and D.

Supplementary Table 5. The Stronger correlations in appendectomy cases between appendectomy-enriched and depleted bacteria

Bacteria-1	Bacteria-2	Rho	Correlation	Transparency
Enterococcus hirae	Enterococcus faecalis	0.608541	Positive	StrongCorr--0.6 <= rho < 0.7
Clostridium sp SY8519	Enterococcus hirae	0.655580	Positive	StrongCorr--0.6 <= rho < 0.7
Bacteroides vulgatus	Aeromonas veronii	-0.220520	Negative	StrongCorr--0.3 <= rho < -0.2
Bacteroides vulgatus	Clostridium sp SY8519	-0.289012	Negative	StrongCorr--0.3 <= rho < -0.2
Bacteroides vulgatus	Blautia sp YL58	-0.273733	Negative	StrongCorr--0.3 <= rho < -0.2
Bacteroides vulgatus	Enterococcus hirae	-0.205366	Negative	StrongCorr--0.3 <= rho < -0.2
Bacteroides vulgatus	Faecalitalea cylindroides	-0.278717	Negative	StrongCorr--0.3 <= rho < -0.2
Bacteroides vulgatus	Enterococcus faecalis	-0.244516	Negative	StrongCorr--0.3 <= rho < -0.2
Bacteroides vulgatus	Anaerobutyricum hallii	-0.268176	Negative	StrongCorr--0.3 <= rho < -0.2
Bacteroides vulgatus	Collinsella aerofaciens	-0.233362	Negative	StrongCorr--0.3 <= rho < -0.2
Bacteroides vulgatus	Blautia sp SC05B48	-0.317485	Negative	StrongCorr--0.4 <= rho < -0.3
Bacteroides thetaiotaomicron	Clostridium sp SY8519	-0.320413	Negative	StrongCorr--0.4 <= rho < -0.3
Bacteroides thetaiotaomicron	Blautia sp YL58	-0.235914	Negative	StrongCorr--0.3 <= rho < -0.2
Bacteroides thetaiotaomicron	Mordavella sp Marseille P3756	-0.250479	Negative	StrongCorr--0.3 <= rho < -0.2
Bacteroides thetaiotaomicron	Lachnospiraceae bacterium Choco86	-0.432693	Negative	StrongCorr--rho < -0.4
Bacteroides thetaiotaomicron	Anaerobutyricum hallii	-0.312155	Negative	StrongCorr--0.4 <= rho < -0.3
Bacteroides fragilis	Enterobacter roggenkampii	-0.208045	Negative	StrongCorr--0.3 <= rho < -0.2
Bacteroides fragilis	Aeromonas veronii	-0.257118	Negative	StrongCorr--0.3 <= rho < -0.2
Bacteroides fragilis	Clostridium sp SY8519	-0.336107	Negative	StrongCorr--0.4 <= rho < -0.3
Bacteroides fragilis	Enterococcus hirae	-0.337307	Negative	StrongCorr--0.4 <= rho < -0.3
Bacteroides fragilis	Enterococcus faecalis	-0.286770	Negative	StrongCorr--0.3 <= rho < -0.2
Bacteroides fragilis	Collinsella aerofaciens	-0.227237	Negative	StrongCorr--0.3 <= rho < -0.2

Supplementary Table 6. The Stronger correlations in controls between appendectomy-enriched and depleted bacteria

Bacteria-1	Bacteria-2	Rho	Correlation	Transparency
Bacteroides fragilis	Bacteroides thetaiotaomicron	0.703902	Positive	StrongCorr--0.7 <= rho < 0.8
Bacteroides vulgatus	Bacteroides fragilis	0.648066	Positive	StrongCorr--0.6 <= rho < 0.7
Blautia sp YL58	Faecalitalea cylindroides	0.807715	Positive	StrongCorr--0.8 <= rho < 0.9
Blautia sp YL58	Mordavella sp Marseille P3756	0.733917	Positive	StrongCorr--0.7 <= rho < 0.8
Faecalitalea cylindroides	Anaerobutyricum hallii	0.810986	Positive	StrongCorr--0.8 <= rho < 0.9
Lachnospiraceae bacterium Choco86	Anaerobutyricum hallii	0.827027	Positive	StrongCorr--0.8 <= rho < 0.9
Mordavella sp Marseille P3756	Anaerobutyricum hallii	0.696953	Positive	StrongCorr--0.6 <= rho < 0.7
Prevotella fusca	Prevotella dentalis	0.859938	Positive	StrongCorr--0.8 <= rho < 0.9
Prevotella ruminicola	Prevotella dentalis	0.884479	Positive	StrongCorr--0.8 <= rho < 0.9
Bacteroides fragilis	Citrobacter freundii complex sp CFNIH3	-0.209527	Negative	StrongCorr--0.3 <= rho < -0.2
Bacteroides thetaiotaomicron	Citrobacter freundii complex sp CFNIH3	-0.210544	Negative	StrongCorr--0.3 <= rho < -0.2
Enterobacter roggenkampii	Eggerthella lenta	-0.215948	Negative	StrongCorr--0.3 <= rho < -0.2
Veillonella dispar	Eggerthella lenta	-0.220393	Negative	StrongCorr--0.3 <= rho < -0.2

Supplementary Table 7. The characteristics of microbiota networks among appendectomy-enriched and depleted bacteria in appendectomy cases

Tax	Degree	Closeness centrality	Betweenness centrality
Bacteroides vulgatus	9	0.088235294	47.96666667
Bacteroides fragilis	6	0.086330935	18.93333333
Bacteroides thetaiotaomicron	5	0.086021505	27.16666667
Clostridium sp SY8519	4	0.087591241	19.33333333
Enterococcus hirae	4	0.086956522	2.8
Enterococcus faecalis	3	0.085714286	1.866666667
Blautia sp YL58	2	0.086021505	5.1
Anaerobutyricum hallii	2	0.086021505	5.1
Aeromonas veronii	2	0.085409253	1.866666667
Collinsella aerofaciens	2	0.085409253	1.866666667
Enterobacter roggenkampii	1	0.082474227	0
Faecalitalea cylindroides	1	0.084210526	0
Mordavella sp Marseille P3756	1	0.082191781	0
Lachnospiraceae bacterium Choco86	1	0.082191781	0
Blautia sp SC05B48	1	0.084210526	0
Veillonella dispar	0	0.04	0
Prevotella ruminicola	0	0.04	0
Bifidobacterium dentium	0	0.04	0
Prevotella fusca	0	0.04	0
Prevotella dentalis	0	0.04	0
Prevotella denticola	0	0.04	0
Citrobacter freundii complex sp CFNIH3	0	0.04	0
Phascolarctobacterium succinatutens	0	0.04	0
Phascolarctobacterium faecium	0	0.04	0
Eggerthella lenta	0	0.04	0

Supplementary Table 8. Significantly differentially enriched species before and after appendectomy in mice.

Species	*P*	Mean Abundance (Before)	Mean Abundance (After)	log2FoldChange (After/Before)	Enrichment
Erysipelotrichaceae bacterium I46	0.047220904	1.00E-09	2.60E-05	14.66742331	After
Shigella sonnei	0.047220904	1.00E-09	2.18E-05	14.41446277	After
Salmonella sp. S13	0.047220904	1.00E-09	1.37E-06	10.42158237	After
Escherichia coli	0.020921335	7.23E-05	0.009764572	7.077852345	After
Bacteroides thetaiotaomicron	0.020921335	0.002033512	0.079584124	5.290435264	After
Bacteroides intestinalis	0.020921335	0.000756087	0.011382064	3.912066417	After
Bacteroides cellulosilyticus	0.020921335	0.000742747	0.011129056	3.905316596	After
Kosakonia arachidis	0.020921335	2.32E-06	2.75E-05	3.565971489	After
Bacteroides xylanisolvens	0.020921335	0.000582105	0.00631284	3.438937594	After
Bacteroides ovatus	0.020921335	0.001166717	0.01015338	3.1214333	After
Brucella ovis	0.013874406	5.58E-07	1.00E-09	-9.124752886	Before
Candidatus Tremblaya princeps	0.047220904	6.32E-07	1.00E-09	-9.302834067	Before
Bacillus sp. DU-106	0.047220904	6.76E-07	1.00E-09	-9.400246906	Before
Campylobacter sp. RM12175	0.013874406	6.85E-07	1.00E-09	-9.419602127	Before
Pseudomonas avellanae	0.047220904	6.92E-07	1.00E-09	-9.433707587	Before
Bacillus pacificus	0.047220904	7.32E-07	1.00E-09	-9.515326192	Before
Borrelia parkeri	0.013874406	8.31E-07	1.00E-09	-9.6979173	Before
Nonlabens sp. MIC269	0.047220904	8.69E-07	1.00E-09	-9.762971882	Before
Staphylococcus sp. AntiMn-1	0.013874406	1.00E-05	1.00E-09	-13.29386436	Before

Supplementary Table 9. SHRs for CRC incidence after PS matching with trimming.

	Total	Appendectomy			Control			Adjusted SHR*	*P*
	NO. of event	NO. of event	Person-years	Incidence rate** (95% CI)	NO. of event	Person-years	Incidence rate** (95% CI)	(95% CI	
CRC	698	310	424,176	73.1 (65.0-81.2）	388	976,844	39.7 (35.8-43.7)	1.730 (1.490-2.010)	<0.001
Age≤50	166	53	292,315	18.1 (13.3-23.0)	113	691,218	16.3 (13.3-19.4)	1.190 (0.848-1.660)	0.320
Age>50	532	257	131,861	194.9 (171.1-218.7)	275	285,626	96.3 (84.9-107.7)	2.020 (1.710-2.396)	<0.001
Female	306	135	234,213	57.6 (47.9-67.4）	171	580,983	29.4 (25.0-33.8)	1.730 (1.380-2.170)	<0.001
Male	392	175	189,963	92.1 (78.5-105.8）	217	395,860	54.8 (47.5-62.1)	1.750 (1.440-2.130)	<0.001
Proximal colon	168	85	422,576	20.1 (15.8-24.4）	83	974,150	8.5 (6.7-10.4)	2.210 (1.640-2.990)	<0.001
Distal colon	237	101	422,688	23.9 (19.2-28.6）	136	974,699	14 (11.6-16.3)	1.670 (1.290-2.150)	<0.001
Rectum	239	101	422,842	23.9 (19.2-28.5）	138	974,521	14.2 (11.8-16.5)	1.570 (1.210-2.020)	<0.001
Unspecific	54	23	422,200	5.4 (3.2-7.7）	31	973,629	3.2 (2.1-4.3)	1.560 (0.914-2.650)	0.100

*Adjusted by Age, Gender, Diabetes mellitus, Hypertension, Hyperlipidemia, diabetes mellitus, hypertension, hyperlipidemia, congestive heart failure, rheumatic disease, dementia, liver disease (divided into mild, moderate, or severe liver disease according to the severity of portal hypertension), myocardial infarction, paraplegia, peptic ulcer, chronic pulmonary disease, chronic kidney disease, and stroke.

**Per 10000 person-years.

CRC: colorectal cancer; CI: confidence interval; PS: propensity score; SHR: sub−distribution hazard ratio.

Supplementary Table 10. Sensitivity analysis on assessing SHRs for cancer incidence after excluding individuals who had a diagnosis of cancer within 3 or 5 years after baseline.

		Number of events			Adjusted SHR (95% CI)	*P*
		Total	Appendectomy	Control		
3 years	CRC	550	227	323	1.560 (1.320-1.850)	<0.001
	female	227	94	133	1.600 (1.220-2.080)	<0.001
	male	323	133	190	1.540 (1.240-1.920)	<0.001
	<=50	146	44	102	1.120 (0.777-1.610)	0.540
	>50	404	183	221	1.852 (1.530-2.248)	<0.001
5 years	CRC	454	180	274	1.490 (1.230-1.790)	<0.001
	female	188	78	110	1.650 (1.230-2.210)	<0.001
	male	266	102	164	1.390 (1.090-1.780)	0.008
	<=50	131	37	94	1.040 (0.697-1.540)	0.860
	>50	323	143	180	1.815 (1.460-2.260)	<0.001

*Adjusted by Age, Gender, Diabetes mellitus, Hypertension, Hyperlipidemia, diabetes mellitus, hypertension, hyperlipidemia, congestive heart failure, rheumatic disease, dementia, liver disease (divided into mild, moderate, or severe liver disease according to the severity of portal hypertension), myocardial infarction, paraplegia, peptic ulcer, chronic pulmonary disease, chronic kidney disease, and stroke.

CRC: colorectal cancer; CI: confidence interval; SHR: subdistribution hazard ratio.

Supplementary Table 11. Primers sequences in validation of the abundance of candidate bacteria by qPCR

Bacteria	Sequences of primers	
Bacteroides vulgatus	Forward	5'-CATGAGTCCGCATGTTCACAT-3'
	Reverse	5'-ACCGTGTCTCAGTTCCAATGT-3'
Bacteroides fragilis	Forward	5'-GGAATGATGTGGAAACATGTCAGTG-3'
	Reverse	5'-GAGTCCTCAGCATAACCTGTTAGTA-3'
Veillonella dispar	Forward	5'- GCGCAACCCCTATCTTATGT-3'
	Reverse	5'-ATTGCTTCCGTCTATTAACTCCC-3'
Enterococcus hirae	Forward	5'-AACGCTTCTTTTTCCACCGGA-3'
	Reverse	5'-TCAAAACCATGCGGTTTCGATTG-3'
Lachnospiraceae bacterium Choco86	Forward	5'-GAATGACCGGTGAGTAATGTCGC-3'
	Reverse	5'- GAGTGCCCATCCTAATTGCTGG-3'
Blautia sp. SC05B48	Forward	5'-AACCGAGTCTTTCCTTCGGG -3'
	Reverse	5'- CTGGCAGTCTCCCCAGAGTG-3'
Total bacteria	Forward	5'-GCAGGCCTAACACATGCAAGTC-3'
	Reverse	5'-CTGCTGCCTCCCGTAGGAGT-3'
